# Heads, Shoulders, Elbows, Knees, and Toes: Modular *Gdf5* Enhancers Control Different Joints in the Vertebrate Skeleton

**DOI:** 10.1371/journal.pgen.1006454

**Published:** 2016-11-30

**Authors:** Hao Chen, Terence D. Capellini, Michael Schoor, Doug P. Mortlock, A. Hari Reddi, David M. Kingsley

**Affiliations:** 1 Department of Developmental Biology, Beckman Center B300, Stanford University School of Medicine, Stanford, California, United States of America; 2 Human Evolutionary Biology, Peabody Museum, Harvard University, Cambridge, Massachusetts, United States of America; 3 Broad Institute of MIT and Harvard, Cambridge, Massachusetts, United States of America; 4 Miltenyi Biotec GmbH, Bergisch Gladbach, Germany; 5 Molecular Physiology and Biophysics and Vanderbilt Genetics Institute, Vanderbilt University, Nashville, Tennessee, United States of America; 6 Center for Tissue Regeneration and Repair, University of California Davis Medical Center, Sacramento, California, United States of America; 7 Howard Hughes Medical Institute, Stanford University, Stanford, California, United States of America; WUSTL, UNITED STATES

## Abstract

Synovial joints are crucial for support and locomotion in vertebrates, and are the frequent site of serious skeletal defects and degenerative diseases in humans. *Growth and differentiation factor 5* (*Gdf5*) is one of the earliest markers of joint formation, is required for normal joint development in both mice and humans, and has been genetically linked to risk of common osteoarthritis in Eurasian populations. Here, we systematically survey the mouse *Gdf5* gene for regulatory elements controlling expression in synovial joints. We identify separate regions of the locus that control expression in axial tissues, in proximal versus distal joints in the limbs, and in remarkably specific sub-sets of composite joints like the elbow. Predicted transcription factor binding sites within *Gdf5* regulatory enhancers are required for expression in particular joints. The multiple enhancers that control *Gdf5* expression in different joints are distributed over a hundred kilobases of DNA, including regions both upstream and downstream of *Gdf5* coding exons. Functional rescue tests in mice confirm that the large flanking regions are required to restore normal joint formation and patterning. Orthologs of these enhancers are located throughout the large genomic region previously associated with common osteoarthritis risk in humans. The large array of modular enhancers for *Gdf5* provide a new foundation for studying the spatial specificity of joint patterning in vertebrates, as well as new candidates for regulatory regions that may also influence osteoarthritis risk in human populations.

## Author Summary

Joints, such as the hip and knee, are crucial for support and locomotion in animals, and are the frequent sites of serious human diseases such as arthritis. The *Growth and differentiation factor 5* (*Gdf5*) gene is required for normal joint formation, and has been linked to risk of common arthritis in Eurasians. Here, we surveyed the mouse gene for the regulatory information that controls *Gdf5'*s expression pattern in stripes at sites of joint formation. The gene does not have a single regulatory sequence that drives expression in all joints. Instead, *Gdf5* has multiple different control sequences that show striking specificity for joints in the head, vertebral column, shoulder, elbow, wrist, hip, knee, and digits. Rescue experiments show that multiple control sequences are required to restore normal joint formation in *Gdf5* mutants. The joint control sequences originally found in mice are also present in humans, where they are marked as active regions during fetal development and post-natal life, and map to a large region associated with arthritis risk in human populations. Regulatory variants in the human *GDF5* control sequences can now be studied for their potential role in altering joint development or disease risk at particular locations in the skeleton.

## Introduction

Synovial joints are the key articulations that connect bones and allow movement in vertebrate skeletons. A typical synovial joint consists of the ends of two long bones, thin layers of articular cartilage covering the bones, a joint cavity filled with synovial fluid, and ligaments and a fibrous capsule that connect the bones and surround the joint. The articular cartilage helps distribute load and provides a smooth lubricated surface to facilitate motion, while the connecting ligaments and capsule provide tethered, guided movement across the joint [1].

Disorders of joint formation and maintenance, including congenital malformations, physical trauma, and degenerative diseases, effect hundreds of millions of people around the world. Most notably, osteoarthritis (OA) in which the articular cartilage and subjacent bone degrade causing substantial joint pain and loss of mobility, affects 13.9% of adults aged 25 and older, and 33.6% of adults aged 65 and older [2]. Despite the prevalence of synovial joint disorders in public health, little is known about the genetic and molecular mechanisms that control joint formation and maintenance, or that control the differential sensitivity of specific joints to risk of OA [3, 4].

Bone morphogenetic proteins (BMPs) are key signaling molecules and receptors known to regulate various aspects of skeletal development [5, 6]. *Growth and differentiation factor 5* (*Gdf5)* was originally isolated as a novel member of this family [7, 8]. Subsequent studies have shown that *Gdf5* is one the earliest known markers of joint formation [9–12]. It is initially expressed in a dramatic pattern of stripes that form 24–36 hours before the histological appearance of the interzone, a region of prechondrogenic mesenchyme fated to become the joint cavity. Subsequently, *Gdf5* expressing cells contribute to many, if not all, adult soft and hard tissue joint structures, including articular cartilage, joint capsule, and ligaments [13, 14].

Genetic studies show that *Gdf5* has conserved roles in normal formation and maintenance of synovial joints in both mice and humans, as well as control of long bone growth. The classic recessive *brachypodism* (*bp*) [15] mutation consists of frameshift changes in the mouse *Gdf5* gene [7]. This viable mutation causes mild reduction of long bone lengths and severe shortening of paws, the latter due to both shorter metapodial bones and loss of one of the three phalanges found in digits rays II-V [16]. *Brachypodism* mutant mice also lack particular synovial joints in the digits, wrists, and ankles [7, 15], form knee joints without the anterior and posterior cruciate ligaments, and develop OA when experimentally challenged [17, 18]. Although *brachypodism* mutants have defects in only a subset of the joints and skeletal structures where *Gdf5* is normally expressed, additional joint and growth defects are revealed in double mutants with other BMP family members, confirming that *Gdf5* also functions in formation of many joints in the limb, sternum, and vertebral column, and in control of multiple skeletal structures [9, 19].

Loss of function mutations in the human *GDF5* gene are also found in patients with acromesomelic chondrodysplasia Hunter-Thompson syndrome [20, 21], Grebe syndrome [22], Brachydactyly Type A2 [23] and C [24, 25]. Some human mutations cause *brachypodism*-like defects at birth, such as the reduction in phalangeal number and the loss of synovial joints. In other syndromes, such as Angel-shaped phalango-epiphyseal dysplasia, hip joint malformations are often coupled with OA [26].

Recent genome-wide and candidate gene association studies show that the genomic interval surrounding *GDF5* can alter risk of common adult OA in humans, even in the absence of obvious congenital skeletal abnormalities. Common variants (minor allele frequency MAF >0.05) spanning a large 130 kb interval from *GDF5* through the downstream gene (*Ubiquinol-Cytochrome C Reductase Complex Chaperone—UQCC*) are significantly associated with a 2-fold increase in hip and knee OA risk [27, 28]. The most studied risk alleles, present in high frequency in Eurasians, are located in the 5' untranslated region (UTR) of *GDF5* [27]. These variants, when tested in constructs containing a *GDF5* minimal promoter region, have been shown to reduce transcriptional activity in articular chondrocyte cells, and are expressed at lower levels than alternate alleles in joint cartilages from total knee replacement patients [27, 29, 30]. No common coding region variants have been identified in previous association studies or in deep exome sequencing projects on OA patients that can explain *GDF5* population level associations with increased OA risk [31–33]. Thus, additional causal regulatory variants may remain undiscovered in the region.

Here, we systematically survey the mouse *Gdf5* locus to map the regulatory sequences controlling expression and function. We identify a large array of non-coding regions that control expression in different subsets of developing joints. Many of the enhancers show surprising specificity for different subsets of joints, due in part to predicted transcription factor binding sites that we show are required for spatial specificity. We find that full functional rescue of mouse phenotypes depends on both upstream and downstream regulatory regions. Strikingly, orthologous regions are found throughout the 130 kb region previously associated with OA, identifying new candidate regions for future functional studies of disease risk in humans.

## Results

### A BAC scan of the *Gdf5* locus reveals upstream and downstream regulatory domains

**Fig 1:**
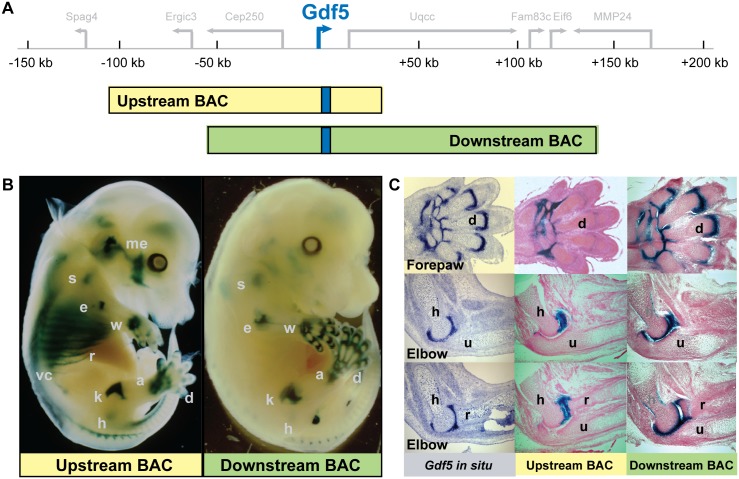
BAC scan of *Gdf5* region reveals both upstream and downstream regulatory domains for joint expression. (**A**) Relative location of two BACs spanning the *Gdf5* region. Upstream BAC (yellow) extends 110 kb upstream to 30 kb downstream of *Gdf5* coding exons, while the Downstream BAC (green) spans an additional 109 kb downstream. IRES-**β**Geo cassette insertion (blue bar) within the 3'UTR of the *Gdf5* coding exons of each BAC generates a visual reporter of *Gdf5* expression. (**B**) Representative transgenic embryos at E14.5 derived from each BAC. Both BACs drive *lacZ* expression in synovial joints of the proximal limb, such as the shoulder (s), elbow (e), hip (h, expression is deep in the tissue), and knee (k); as well as the distal limb, such as the wrist (w), ankle (a), and digit joints (d). Additionally, expression was detected in axial tissues such as the ribs (r), in and around vertebral column (vc), and some sites in the head, such as the middle ear (me). In general, the Upstream BAC drove stronger proximal limb expression while the Downstream BAC drove stronger distal limb expression. (**C**) E14.5 embryonic forepaw and elbow sections comparing the endogenous *Gdf5* expression pattern *via in situ* hybridization (left) with the *lacZ* patterns driven by the Upstream (middle) and the Downstream BACs (right). Note that the endogenous *Gdf5* gene is expressed in stripes at most sites of joint formation, including both the humeroulnar (h-u) joint and the humeroradial (h-r) joints of the elbow. The Upstream BAC drives expression in many proximal limb joints, but only in the humeroradial and not the humeroulnar joint of the elbow. In contrast, the Downstream BAC drives comprehensive appendicular joint expression, including both joints of the elbow. All expression patterns were confirmed in multiple independent transgenic embryos (S1 Table).

To identify *cis*-regulatory elements precisely controlling *Gdf5* expression in joints, we initially performed a BAC scan encompassing a 250 kb genomic interval centered on the mouse *Gdf5* locus. We first chose a BAC that covers 110 kb upstream to 30 kb downstream of the *Gdf5* coding region and modified it by inserting an Internal Ribosome Entry Site (IRES)-βGeo reporter cassette into the gene’s 3'UTR ("Upstream BAC", Fig 1A). The IRES reporter cassette allows dicistronic translation of both the GDF5 and LacZ proteins from *Gdf5* mRNA. We then tested this construct’s ability to drive *lacZ* expression in multiple independent transgenic mouse embryos at embryonic day 14.5 (E14.5), a key period when synovial joints form. The Upstream BAC drove consistent expression in axial and appendicular sites characteristic of the endogenous *Gdf5* expression pattern (Fig 1B). In the axial skeleton, *lacZ* was detected in ribs, the vertebral column, and in head structures such as the middle ear and mandible (Fig 1B) among other sites (S1 Fig). Each of these sites corresponds to locations where the endogenous *Gdf5* gene is also expressed (S1 Fig). In the limb, *lacZ* expression was found in proximal synovial joints of the shoulder, elbow, hip, and knee, as well as distal joints of the wrist, ankle, and digits, albeit expression was both lower and less reproducible in these distal domains (S1 Table). Serial sections of X-gal stained limbs showed that extensive *lacZ* expression was present in the wrist and ankle regions, with weaker signal in the digit joints, especially compared to the endogenous *Gdf5* expression (Fig 1C, top panel). In the elbow region, whereas endogenous *Gdf5* gene expression was detected in both the humeroradial (h-r) and humeroulnar (h-u) joints, *lacZ* was detected only in the humeroradial joint (h-r) (Fig 1C, middle and bottom panels). Therefore, while the Upstream BAC contains key regulatory elements that regulate *Gdf5* expression in some joints, it appears to lack regulatory sequence that can faithfully recapitulate the complete *Gdf5* expression pattern.

To expand our search for additional joint control elements, we isolated a second BAC that extends ~110 kb further downstream of our first BAC, and again inserted an IRES-βGeo reporter cassette into the same position in the *Gdf5* 3'UTR ("Downstream BAC", Fig 1A). In E14.5 transgenic mouse embryos, this Downstream BAC drove striking *lacZ* expression in all proximal synovial joints of the shoulder, elbow, hip, and knee, as well as in distal joints of the wrist, ankle, and digits (Fig 1B). It additionally drove expression in the same axial and head structures as the Upstream BAC, albeit at much weaker levels, possibly because of repressor sequences also present in the Downstream BAC interval or positional effects of BAC transgene integration (Fig 1B, S1 Table). Compared to the Upstream BAC pattern, *lacZ* expression was much stronger in the transverse joint stripes of the digits and encompassed a wider domain that included the mesenchyme of the inter-digital space (IDS) and each digit’s lateral and medial border (Fig 1C, top panel). Further inspection revealed that, unlike the Upstream BAC, the Downstream BAC drove *lacZ* expression across both the humeroradial (h-r) and humeroulnar (h-u) joints in the elbow (Fig 1C, middle and bottom panels). Overall, the *lacZ* expression pattern produced by the Downstream BAC closely recapitulated the endogenous *Gdf5* expression pattern in limbs.

### Downstream and upstream BAC sequences continue to drive strong joint expression in adulthood

We further assessed the regulatory control of *Gdf5* at later stages of joint development when joint structures, such as tendons, ligaments, articular capsules, and articular cartilages mature. In metapodial-phalangeal joints, intervertebral joints, as well as the calcaneal insertion site of the Achilles tendon, the Upstream BAC continued to drive *lacZ* expression in adult animals (S2 Fig). In the knee at E17.5 we found that the Downstream BAC drove strong *lacZ* expression in nearly all joint tissues and surfaces, whereas the Upstream BAC drove weak expression in very restricted domains such as the most inferior and superior articular surfaces of the femur and tibia, respectively, as well as in the developing cruciate ligaments (S3 Fig). The strong knee expression exhibited by the Downstream BAC continued during post-natal development and through adult joint maintenance stages (S3 Fig). Conversely, Upstream BAC sequences failed to drive detectable *lacZ* expression via X-gal staining techniques in the knee during adulthood (S3 Fig). These data reveal that the upstream and downstream regions contain enhancers active during both early joint formation, and in joint homeostasis during post-natal life. Such expression is expected, since endogenous *Gdf5* expression has also been detected in the adult articular cartilage and joint tissues of both mice and humans [29, 34, 35].

### Two separable upstream sequences of *Gdf5* drive expression in axial structures and proximal synovial joints

**Fig 2:**
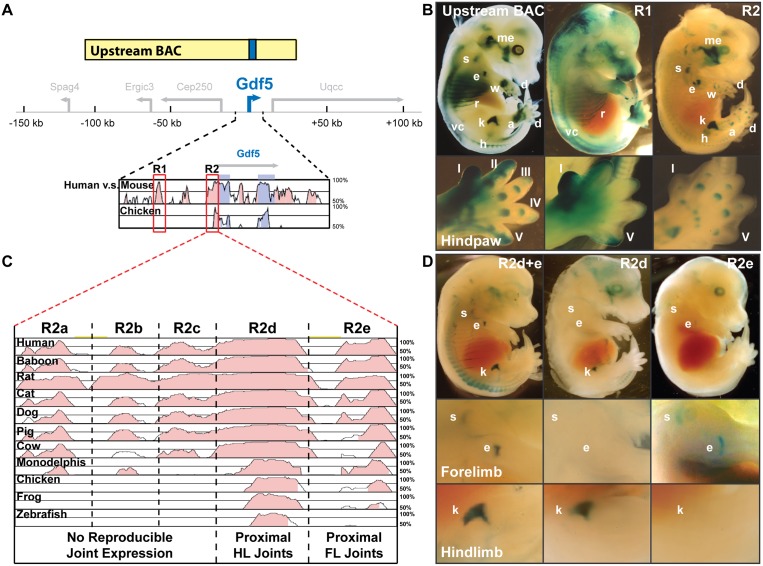
Two upstream regulatory elements drive expression in axial tissue domains (R1) and limb joints (R2); while separate sub-domains of R2 control distinct positions with limbs. (**A**) Evolutionarily conserved non-coding regions (pink peaks) were identified upstream of *Gdf5* coding regions (blue peaks), based on a criterion of 70% or greater nucleotide sequence identity between human, mouse, and chicken sequence over a region of 300 bp or more. (**B**) Two distinct conserved non-coding peaks (R1 and R2; red-boxes in **A**) were tested for enhancer activity at E14.5 and compared to *lacZ* expression driven by the Upstream BAC. R1 drove expression in axial tissues such as the ribs (r), vertebral column (VC), and anterior mesenchyme of the paw, while R2 drove expression in limb joints such as the shoulder (s), elbow (e), wrist (w), hip (h), knee (k), ankle (a), and digit regions (d). Bottom panel shows zoom-in images of hindpaws. (**C**) Comparison of genomic sequences of R2 from twelve species with mouse as reference. Based on patterns of conservation between species, R2 was divided into five sub-regions (a-e). (**D**) Transgenic embryos at E14.5 from sub-regions R2d+e (left), R2d (middle), and R2e (right). Sub-region R2d drove hindlimb (HL) joint expression, while sub-region R2e drove forelimb (FL) joint expression. Panels below show zoom-in images of forelimb (shoulder, elbow) and hindlimb (knee) from R2d+e, R2d, and R2e embryos.

To further identify specific non-coding sequences controlling different anatomical patterns, we first took advantage of the finding that the expression pattern of *Gdf5* is well conserved between humans [29], mice [9], and chicken [11]. We used VISTA and PIP maker (Methods) to identify multiple non-coding regions that were also well conserved across species (Fig 2A). We then cloned different non-coding conserved regions upstream of a minimal promoter and *lacZ* reporter, and tested the ability of different constructs to drive specific patterns in E14.5 mouse embryos.

Two regions, contained within the upstream *Gdf5* domain shared by both BACs, drove relevant expression patterns (Fig 2A, red boxes). Region R1 (754 bp), located 5 kb upstream of *Gdf5*, controlled *lacZ* expression in many of the axial sites previously described for each BAC (Fig 2B). These domains include the ribs, the vertebral column, and sites within the head and jaw (S1 Table). This sequence also drove wide expression in the mesenchyme of the anterior interdigital space of the fore- and hindpaw (Fig 2B). Region R2 (802 bp), adjacent to the promoter region of *Gdf5*, drove consistent *lacZ* expression in the proximal synovial joints of the shoulder, elbow, hip, and knee (Fig 2B; S1 Table). However, as observed with the Upstream BAC, this element did not drive expression in the humeroulnar (h-u) joint (Fig 2B and S4B Fig, bottom panels) and expression in the digital joints was weak and less reproducible (Fig 2B). Importantly, these two evolutionarily conserved non-coding elements when considered *in toto* drove *lacZ* in a pattern closely resembling the Upstream BAC (Fig 2B).

### Different subregions of the R2 upstream element drive preferential expression in forelimb or hindlimb joints

To further delineate specific joint expressing domains within the R2 regulatory region, we used truncation mapping in the context of a comparative genomic sequence analysis. Sequences from twelve different species (human, baboon, mouse, rat, cat, dog, pig, cow, opossum, chicken, frog, and zebrafish) were precisely aligned using Vista (Fig 2C) to identify conserved sub-regions within R2. Analysis of sequence conservation across this element revealed at least 5 sub-regions of weak to strong conservation (R2a-e). While sub-regions R2a+b+c were weakly conserved; sub-region R2d was particularly well conserved across a diverse set of placental mammals, opossum, chicken, and zebrafish; and sub-region R2e was conserved to frog. Given these differences in conservation, sub-regions R2a+b+c and R2d+e were cloned separately into our *lacZ* reporter system and tested for their expression in E14.5 transgenic embryos. Sub-regions R2a+b+c did not drive any reproducible pattern of *lacZ* expression (S1 Table). On the other hand, sub-regions R2d+e together drove *lacZ* expression in the proximal limb joints of the shoulder, elbow, hip, and knee, but not in distal digit joints (Fig 2D). Interestingly, in the elbow joint, sub-region R2d+e drove *lacZ* in the humeroradial articulation only (Fig 2D), a restricted pattern identical to that observed for the complete R2 element (Fig 2B and S4B Fig) and the Upstream BAC (Fig 1B and 1C).

To further examine the regulatory capacity of sub-regions R2d versus R2e, smaller constructs containing each (i.e., R2d or R2e) were individually tested in our *lacZ* reporter assay in transgenic embryos. Sub-region R2d drove *lacZ* expression only in the proximal joints of the hindlimb (i.e. hip and knee) (Fig 2D, R2d). In contrast, sub-region R2e regulated *lacZ* expression only in the proximal joints of the forelimb (i.e. shoulder and elbow) (Fig 2D, R2e). These data provide striking evidence that expression in particular fore- and hindlimb joints can be regulated by separate DNA elements.

### Predicted transcription factor binding sites are required for expression in particular joints

**Fig 3:**
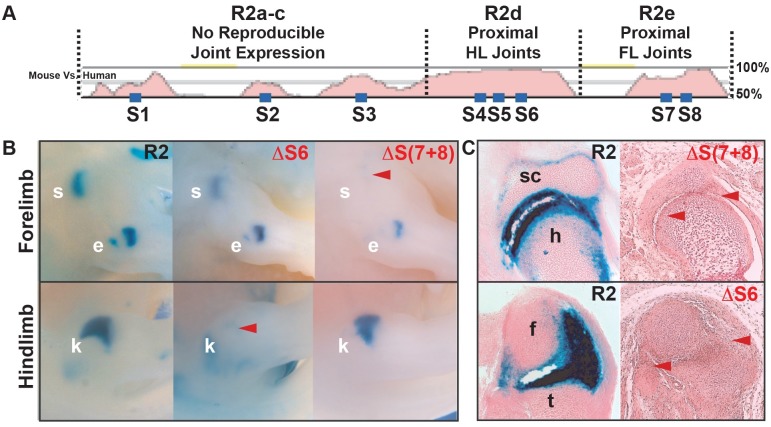
Predicted homeodomain binding sites are required for R2 expression domains in specific joints. (**A**) Eight predicted homeodomain binding sites (S1-S8, blue boxes) are found across R2 subregions. Four of these UNIPROBE predicted sites are found in both mice and humans, including S2 in sub-region R2a-c, S5 and S6 in sub-region R2d, and S8 in sub-region R2e. (**B**) Targeted mutagenesis of conserved S6 within the hindlimb regulatory zone of R2 eliminates normal *lacZ* signal in the knee (red arrowhead) but not elbow or shoulder, tissues where this enhancer region is predicted to have little to no influence on expression. Mutagenesis of sites S(7+8) within the forelimb regulatory zone of R2 eliminates expression in shoulder (red arrowhead) but not elbow or knee, tissues where this sequence is predicted to have little to no influence on expression. (**C**) Histology at E14.5 of wild-type R2 (left) versus mutant R2 (right) constructs reveals specific reductions (red arrowheads) in joint domains for each construct. Abbreviations: s, shoulder; e, elbow; k, knee; sc, scapula; h, humerus; f, femur; t, tibia.

To look for possible factors that may contribute to joint-specific patterns, we used two different programs to identify putative transcription factor binding sites in the R2 element, the UNIPROBE database [36] and rVista/MatInspector [37–40]. At the recommended enrichment of 0.4, UNIPROBE predicted binding sites for a number of different transcription factors (S2 Table), of which, many are homeodomain proteins that overlap in their binding site preferences. One of these potential upstream factors, BARX, is a homeodomain protein with known roles in chondrogenesis [41]. *In situ* hybridization studies in mouse and chick reveal that BARX1 and BARX2 family members remarkably overlap in expression with *Gdf5* in proximal and distal joints [41–44]. Given the known roles of BARX2 in chondrogenesis in the mouse, we used the BARX motif to further search for conserved homeodomain binding sites between humans and mouse. We identified 8 predicted BARX2 sites (S1-S8) in the mouse R2 region at a UNIPROBE enrichment cutoff of 0.4. Four of these predicted sites are mouse-specific (S1, S3, S4, and S7), and four are additionally conserved using the same UNIPROBE enrichment cutoff in the human R2 ortholog (S2, S5, S6, and S8) (Fig 3A; Methods). Note that because of overlap of the binding specificities of many homeodomain containing factors, these sites could also represent binding sites for other factors besides BARX proteins.

We next wanted to test *in vivo* whether any of the predicted homeodomain (e.g., BARX2) binding sites are required for *lacZ* expression. Since sites S1-S3 lie in a R2 sub-region that yielded no reproducible enhancer activity, we concentrated on sites S4-S6 in the hindlimb (hip/knee) sub-region, and on S7-S8 in the forelimb (shoulder/elbow) sub-region. Using a database of experimentally measured binding interactions between many vertebrate transcription factors and all possible 8-mer target sequences (UNIPROBE, Methods), we found that changing 3 or 4 bases in the predicted BARX2 site was sufficient to reduce experimentally determined BARX2 binding to values below significance (P>0.05) and enrichment (E<0.4) thresholds (S3 Table, Methods). We therefore generated new constructs containing the R2 enhancer with different predicted BARX2 sites modified, and tested for effects on expression patterns in transgenic mouse embryos at E14.5. While mutation of individual sites S4 or S5 had no observable effects, mutation of S6 eliminated expression within the knee (Fig 3B), but not the hip (S1 Table) in multiple transgenic lines tested. Histological analysis revealed that *lacZ* expression was absent from the capsule and articular joint surfaces in the knee. Mutation of S7 had no visible effect, while mutation of S8 slightly reduced scapulo-humeral expression (S1 Table). However, mutation of both S7 and S8 sites completely eliminated expression only within the shoulder but not elbow indicating that these two sites may cooperate to drive expression in the scapulo-humeral joint (Fig 3B). Finally, we also mutated two additional binding sites identified by MatInspector (PITX1 and ZEB1) and found no significant visible expression changes in shoulder, knee, and elbow compared to control constructs (S5 Fig, S1 Table).

### Downstream regulatory sequences also contain multiple limb enhancers

**Fig 4:**
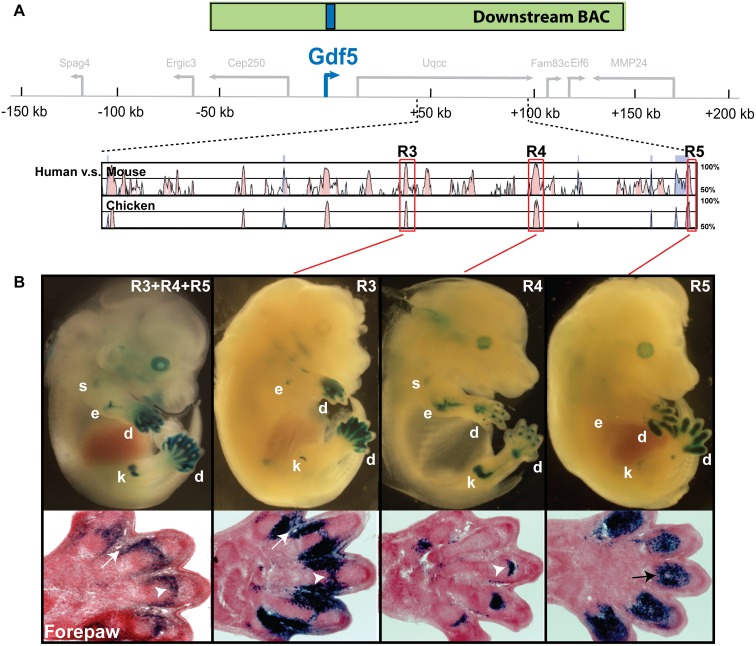
A cluster of three downstream regulatory enhancers interact to control gene expression in distinct distal limb tissues. (**A**) Three evolutionarily conserved elements (red boxes) within the Downstream BAC (green) region were identified via a comparison of mouse, chicken, and human sequences. Pink peaks represent conserved non-coding sequence showing at least 70% nucleotide identity over a 300 bp window, while blue peaks reflect conservation in coding sequence. (**B**) Transgenic embryos collected at E14.5 from constructs containing either all three conserved non-coding elements as a concatenate (R3+R4+R5) (left) or individual regulatory elements (R3, R4, or R5 as indicated). Panels below each embryo are histological sections of the forepaw digital domains. When combined, all three elements (R3+R4+R5) drive weak expression in proximal limb joints such as the shoulder (s) and strong expression in the distal limb joints, such as the elbow (e), knee (k), and digits (d), along with the interdigital space (white arrow). R3 by itself drives expression in distal joints but also strongly in the interdigital space (white arrow) and interphalangeal joint (white arrowhead); R4 drives expression in distal limb joints including the interphalangeal joint (white arrowhead); and R5 drives expression weakly in distal limb joints, yet strongly in the phalangeal mesenchyme of the digits (black arrow).

Since the Upstream BAC was not able to drive strong gene expression in distal joints, or in specific joints of the mid-limb, we also searched for additional non-coding sequences that were present in the Downstream BAC region (Fig 4A). We identified three non-coding regions evolutionarily conserved to chicken and located far downstream of *Gdf5*: R3 of 586 bp at +71 kb relative to the mouse *Gdf5* coding region, R4 of 975 bp at +81 kb, and R5 of 337 bp at +98 kb (Fig 4A, red boxes). We tested each in our transgenic assay at E14.5. R3 drove *lacZ* expression in the mesenchyme of the interdigital space and in digital transverse joint stripes as well as in the elbow and knee (Fig 4B, R3). R4 drove very strong expression in the joints of elbow, knee, digits, with the expression in the joints of shoulder and hip considerably weaker (Fig 4B, R4). R5 drove strong *lacZ* expression in the pre-chondrogenic mesenchyme of developing phalanges (Fig 4B, R5), and very weakly in the elbow and knee. One additional conserved region (GROW1 at +45 kb) drove expression in growth collars at ends of long bones rather than in developing joints. The possible role of GROW1 in controlling lengths of long bones is the subject of a separate manuscript (Capellini, Chen et al., in review).

The unique phalangeal pre-chondrogenic mesenchymal expression driven by R5 was not visible in embryos from the Downstream BAC construct, which covers both R5 and the other conserved regions tested above (Figs 1 and 4B). To explore this observation further, we concatenated R3, R4, and R5 and tested them as a single construct. Interestingly, we found they controlled expression in the same pattern as the Downstream BAC, i.e., within proximal limb joints of the shoulder, elbow, hip, and knee as well as distal joints of digits (Fig 4B, R3+R4+R5). As shown by histological sections, R3+R4+R5 collectively drove strong expression in the transverse stripes of the distal digit joints, along the lateral and medial joint capsules, and within the interdigital space (Fig 4B, arrows). However, the overall digit mesenchymal expression pattern of R5 was not reproduced by this construct, suggesting that R3 or R4 may act as a repressor element for the digit mesenchyme expression of R5. The possible trans-acting factors mediating this repressive effect are still unknown, but could be searched for in the future once the cis-acting elements are further narrowed. We also note that the overall expression driven by the composite R3+R4+R5 construct was both stronger and more reflective of the complete *Gdf5* expression pattern than the pattern driven by the Upstream BAC (Fig 1B and 1C). Sections through the elbow joint indicate that the R3+R4+R5 construct drove gene expression in both the humeroradial (h-r) and humeroulnar (h-u) joints, consistent with the pattern of R4 (S4 Fig) and the Downstream BAC, but unlike the Upstream BAC (Fig 1B and 1C). Therefore, these three evolutionarily conserved non-coding regions located far from *Gdf5* contribute to additional sites of expression in joints and long bones (S1 Table). Several of these expression sites are not seen with Upstream control elements, while others are not readily apparent by testing only large genomic sequences (i.e., BACs).

### Partial or complete rescue of *Gdf5* mutant phenotypes by upstream and downstream BAC sequences

To test the functional capacities of upstream and downstream regulatory domains, we took advantage of the fact that functional copies of the *Gdf5* coding region are present within both of the Upstream and Downstream BAC clones. Transgenic mice carrying each BAC were bred onto a *brachypodism (bp)* mutant background, followed by detailed examination of anatomical sites typically altered in *Gdf5* mutant animals.

**Fig 5:**
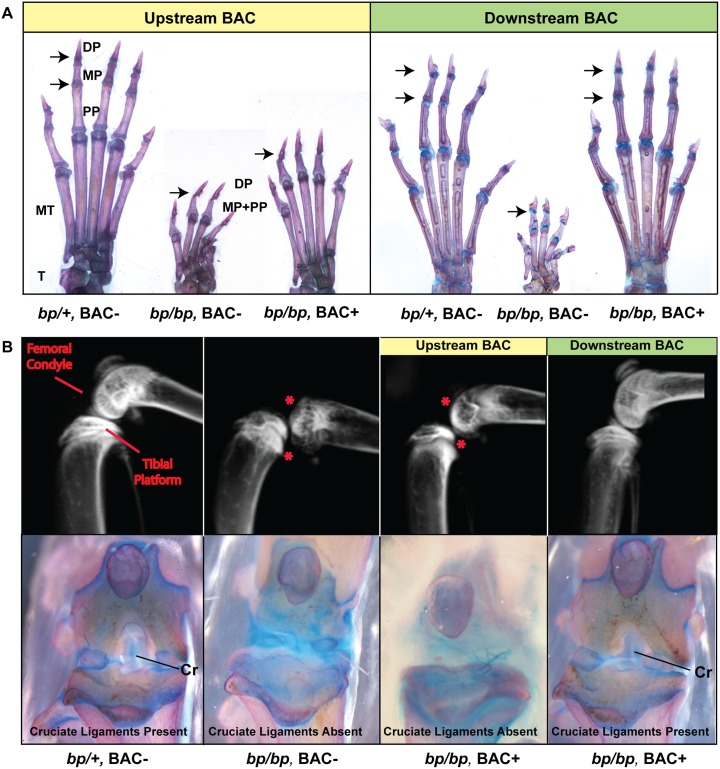
Downstream *Gdf5* regulatory regions are required for full rescue of *brachypodism* joint defects. (**A**) Skeletal preparations of hindpaws of transgenic rescue experiments with either Upstream (yellow) and Downstream (green) BACs. Control mice (*bp/+*, *BAC-*; left) are shown in comparison to *brachypodism* homozygous mutants that are either BAC negative (*bp/bp*, *BAC-*; center) or are also carrying either Upstream or Downstream BAC positive (*bp/bp*, *BAC+*; right). In each panel digit I is to the right. DP (distal phalange), MP (middle phalange), PP (proximal phalange), MT (metatarsal), and T (tarsal). Arrows indicate location of the interphalangeal joints. (**B**) (Top) Radiographs of adult mouse knee joints. Asterisks (*) indicate the femoral and tibial dysmorphologies typical of *bp/bp* mutants. (Bottom) Alcian Blue (cartilage) and Alizarin red (bone) preparations of adult knees from rescue experiments. Cruciate ligaments (Cr) are usually missing from *bp/bp* mice. Note that the presence of the Downstream BAC (*bp/bp*, *Downstream BAC+* animals) fully restores interphalangeal joints, adult knee joint articular morphology, and cruciate ligaments; while the Upstream BAC does not.

Hindlimbs of *brachypodism* homozygous mutants usually show fusions of tarsal bones or supernumerary bony elements in the ankle region, reduced length of metatarsals, absent middle phalanges on digits II-V, and much shorter digits (Fig 5) [7, 15]. The presence of the Upstream BAC restored normal formation in tarsal joints and ankles, and improved metatarsal lengths compared *bp/bp* mutants (Fig 5A, left). However middle phalanges were missing in Upstream BAC-positive mice, and digits were still substantially shorter than wild type. Thus, normal bone and joint patterning in digits is not rescued by Upstream BAC sequences.

In contrast, the Downstream BAC sequences fully restored multiple phenotypes in *bp/bp* mutants. BAC-positive mice showed normal tarsals and ankles, metatarsal and phalangeal lengths, and possessed the middle phalanges in digits II-V (Fig 5A, right).

In addition to possessing dysmorphic and shorter paws than wild-type mice, *bp/bp* mutants develop knee OA when experimentally challenged [18] and consistently display dislocated joints with poorly developed femoral and tibial articular surfaces and absent cruciate ligaments (Fig 5B) [17]. The Upstream BAC partially restored distal femoral condylar morphology but was unable to restore the proper registration of the femur and tibia, or rescue the absence of the anterior and posterior cruciate ligaments (Fig 5B). In contrast, the Downstream BAC allele rescued multiple knee phenotypes, leading to proper registration between femur and tibia, normal morphology of joint surfaces, and restored cruciate ligaments (Fig 5B). Thus, although Upstream BAC sequences can partially rescue some *Gdf5* phenotypes, including normal joint formation in ankle regions, additional sequences in the Downstream BAC are required to rescue joint formation in distal digit regions, and to restore knee surfaces and ligaments.

## Discussion

We have identified a series of *cis*-acting regulatory elements distributed upstream and downstream of the *Gdf5* gene spanning an approximate 100 kb interval in mice. Previous studies showed that an upstream BAC clone could recapitulate multiple aspects of *Gdf5* expression, including expression in multiple joint interzones [13]. Although the upstream BAC clone can be used to drive Cre expression and modify the activity of other genes in joints [13], additional downstream regulatory sequences are clearly required to achieve full expression in limbs, to fully rescue normal bone and joint formation in digits, and to restore knee structures in *brachypodism* mutant mice.

**Fig 6:**
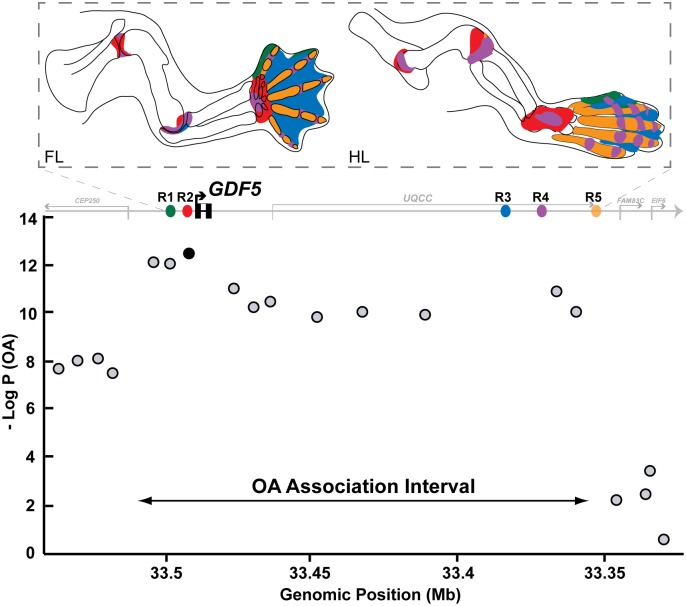
Modular regulatory architecture of *GDF5* spans the region linked to Osteoarthritis (OA) susceptibility in humans. (Top) Summary of the different stripes and anatomical domains controlled by separate regulatory enhancers (colored) in the *Gdf5* gene in E14.5 developing mouse forelimbs (FL) and hindlimbs (HL). (Bottom) Association of various human SNPs (grey circles) with adult knee OA in cases vs. controls (based on [27]). Y-axis is the -log P-value of the trait association for SNPs across the interval. X-axes show genomic megabase locations (bottom axis) of human sequences orthologous to R1 (green), R2 (red), R3 (blue), R4 (purple), and R5 (orange) elements (top axis). The highest scoring variant tested in the human study, rs143383 (dark circle), is located in *GDF5* 5'UTR, immediately downstream of the R2 region. Note that significant association extends over a broad region, and many linked human variants have not yet been tested, including common human variants in R2, R3, R4, and R5 (see S4 Table).

Separable enhancers within both the upstream and downstream regions show a striking degree of anatomical specificity, including: 1) different anatomical divisions within the body (e.g., appendicular skeleton, axial skeleton, cranium); 2) different skeletal tissue types (e.g., articular cartilage, interdigital mesenchyme, pre-cartilaginous mesenchyme); 3) different limb types (e.g., forelimb, hindlimb); 4) different domains within the limb (e.g., proximal limb, distal limb); and 5) different individual joints within limbs (e.g., elbow versus knee; humeroradial versus humeroulnar joints) (Fig 6, upper panel; S1 Table). If and how these sequences regulate additional genes in their vicinity is currently unknown. We note, however, that the *centrosomal protein 250KD* (*Cep250*) gene and the *ubiquinol-cytochrome c reductase complex assembly factor 1* (*Uqcc1*) genes, which map upstream and downstream of *Gdf5*, do not show the striking joint expression patterns that are shared by *Gdf5* and the various joint enhancers reported here (S6 Fig).

In terms of overall regulatory architecture, our results are reminiscent of previous studies of repeating stripes of gene expression during early Drosophila development. In Drosophila, multiple pair-rule genes are expressed in seven characteristic stripes in early embryos [45]. For "secondary" pair rule genes, transcriptional control of the seven-stripe pattern depends upon on earlier "primary" pair rule genes. In contrast, transcriptional control of "primary" pair rule genes like *hairy* and *even-skipped* (*eve*) is considerably more complex [46, 47]. For example, multiple distinct enhancers in the *eve* locus control expression only in particular stripes or subsets of stripes, with specific enhancers responding to different types and concentrations of upstream transcription factors that are found at particular anatomical positions along the anterior-posterior axis [48]. The simple appearing multiple-stripe pattern of the *eve* locus is thus built from a composite of underlying mechanisms, and several different enhancers within the gene are required to reproduce the overall pattern [49].

Our results suggest that the vertebrate *Gdf5* locus is controlled in a similar piecemeal fashion. *Gdf5* is expressed in a striking pattern of stripes that are seen almost everywhere that synovial joints form in the head, vertebral column, and limbs (Fig 6, upper panel). Although the regularity of the stripe expression in interzones might have been controlled by a common regulatory mechanism, different joint stripes are clearly controlled by separate regulatory enhancers which are distributed over large genomic regions both upstream and downstream of *Gdf5* coding sequences. At least one additional enhancer in the *Gdf5* downstream region is expressed in collars near the ends of bones, and may be involved in regulating long bone growth rather than joint formation or maintenance (Capellini, Chen et al., in review). Multiple enhancers are required not only to recreate the gene’s overall expression pattern in embryos, but also to rescue limb phenotypes seen in *Gdf5-*deficient mice, confirming the importance of the distributed *cis*-acting control sequences for overall *Gdf5* function.

Unlike the case in Drosophila, the upstream factors that regulate expression from individual *Gdf5* enhancers are still largely unknown. Multiple binding sites can be predicted in the sequences of the R1, R2, R3, R4, and R5 elements identified in this study, based on large databases of binding interactions between transcription factors and target sequences (UNIPROBE, [50]), as well as large-scale chromatin-immunoprecipitation (ChIP-seq) experiments from cell lines and embryos. However, interzones are transient structures that are not recapitulated in immortal cell lines. They also make up only a small fraction of cells in developing embryonic structures, and so are not well represented in bulk studies of transcription factor binding in developing tissues. Given the relatively large size of the *Gdf5* enhancers as defined by functional studies, the small size of most consensus binding sites, and the relative paucity of interzone cells in most large-scale chromatin interaction studies, many relevant factors controlling specific stripes of *Gdf5* expression likely remain to be discovered.

Despite these difficulties, some candidate upstream regulators can be postulated, based on genes that are required for normal joint development, or that are differentially expressed in particular limbs or different joints within the limb, including WNT signals, and members of the SOX, HOX, TBX, DLX, PITX, and ZEB1 transcription factor families [51–65]. Predicted PITX1 and ZEB1 binding sites are present in the R2 enhancer element, and the known hindlimb-specific expression of PITX1 would appear to be a promising mechanism for controlling hindlimb-specific stripes of *Gdf5* expression. However, mutating either PITX1 or ZEB1 binding sites did not significantly alter R2d+e enhancer joint patterns in the limbs (S5 Fig, S1 Table), suggesting that additional binding sites or other upstream factors are likely involved.

Members of the BARX family of transcription factors are also expressed in interzone stripes, a pattern that closely resembles that of *Gdf5*. *Barx2* null mice show altered joint formation but do not lose *Gdf5* expression [41]. However, *Barx1* is also expressed in limbs and joints [66, 67], and functional redundancy among BARX family members may make it difficult to detect the full range of *Barx* phenotypes in developing joints. Given the overlapping expression patterns of *Barx2* and *Gdf5*, we tested the effects of mutating specific BARX2 binding sites in the R2 enhancer, though the predicted sites may also bind additional factors. Individual mutation of some sites (i.e., S4, S5, S7) had no visible effect on joint-specific enhancer activity as assayed by *lacZ* expression. However, mutation of site S6 significantly reduced expression within the knee, whereas mutations of both S7 and S8 reduced shoulder expression. It is possible that our site-specific mutations disrupt binding sites for other upstream factors in addition to BARX2, including other homeodomain factors that bind similar sequences. Nonetheless, our results clearly identify specific small sequence motifs within the R2 enhancer that are required for expression in individual joints in forelimbs or hindlimbs. Future studies of these and other enhancer sequences should help elucidate mechanisms controlling expression in specific joints in the vertebrate skeleton.

### Evolution of *Gdf5* control sequences

*Gdf5* is expressed in synovial joints from a wide range of species, including fish, birds, and mammals [9, 11, 21, 29, 68, 69]. Comparative sequence analysis shows that the 5' R2 regulatory element controlling strong proximal limb joint expression is conserved from mammals to at least *Danio rerio* (Zebrafish), while the 3' distal limb element cluster (R3+R4+R5) is conserved only between mammals and *Xenopus tropicalis* (Western clawed frog). The 5' joint control element may be older (or more constrained) than the joint elements in the 3' distal cluster, perhaps because R2 is required to build the more ancient proximal fin skeleton of aquatic vertebrates. Conversely, the distal 3' regulatory elements, which control expression not just in joints but in lateral/medial chondrogenic tissues, interdigital space, and developing phalangeal element mesenchyme, may have evolved during the later emergence of distal limb structures in terrestrial vertebrates. Such additive modular control has been posited for other organs. For example, *Nkx2-5* has multiple distinct *cis*-acting elements that direct transcription specifically in different sub-regions of the developing heart [70–73] and this complexity may have played an important role in the evolution of a multi-chambered mammalian heart [74].

Modular regulatory architectures may be particularly important for genes that play roles in the development of highly patterned structures that have distinct sizes and shapes at particular anatomic positions in different species, such as skeletons and muscles. Other BMPs involved in cartilage and bone formation, such as *Bmp5* and *Gdf6* [75–77] as well as members of the *MyoD* and *Mrf* families that control muscle determination [78, 79] are all controlled by complex sets of highly specific modular enhancers. Gain, loss, or modification of anatomically specific enhancers within such genes may be a common way of altering particular structures in the body during evolution, while preserving other essential aspects of gene function [80–83].

### Implications for human health and disease

Multiple human congenital skeletal defects have previously been traced to coding region changes in the *GDF5* gene, including: acromesomelic chondrodysplasia Hunter-Thompson syndrome [20, 21], Grebe syndrome [22], Brachydactyly Type A2 and C and synostoses [84, 85].

More recently, common forms of OA have also been linked to genetic variation around the *GDF5* locus, in the absence of protein coding changes. Miyamoto et al. (2007) was the first to report that derived SNP variants “T” at rs143383 and “T” at rs143384 in the 5'UTR region of *GDF5* are significantly associated with common hip and knee OA in Japanese and Chinese populations (1.5–2.1 OR) [27]. This finding was confirmed in Europeans [29], and many subsequent studies have reported significant associations in Eurasians between the same SNPs and OA in different joints such as the hand, hip, and knee (selected examples include [86–92]).

Reconstruction experiments have shown that the derived “T” risk alleles at rs143383 and rs143384 reduce quantitative levels of gene expression when transfected with reporter genes into tissue culture cells *in vitro* [27, 33]. Additionally, a rare *cis*-acting promoter variant has been shown to modulate the activities of both “T” variants in similar transfection assays [33]. Although both common “T” variants are actually located in the transcribed 5'UTR region of *GDF5*, they are closely linked to the R2 upstream enhancer region identified here. To test for possible qualitative effects of these SNPs on joint expression patterns *in vivo*, we cloned both the derived "T,T" risk variants and the ancestral "C,C" protective variants of rs143383 and rs143384 into separate 1468 base pair constructs carrying the R2 enhancer upstream of the Hsp68 basal promoter and a *lacZ* expression cassette. We did not observe any visible difference in expression within the different joints of the limb when comparing transgenic mouse embryos made with the different constructs (S7 Fig, S1 Table). Subtle expression differences would be difficult to detect with this assay, and it possible that more substantial differences would be detected at different time points or in the presence of other disease associated mutations.

Interestingly, the genomic region significantly associated with OA risk in humans actually extends substantially beyond the rs143383 and rs143384 SNPs in the 5'UTR of the gene (Fig 6, bottom panel) [27]. A 130 kb risk haplotype is present at high frequency in Eurasian populations. Markers throughout this region are in high linkage disequilibrium with the 5'UTR SNPs, and show similarly high association with OA phenotypes in studies that have tested additional markers [27, 86–92]. We note that the human orthologs of the R2, R3, R4, and R5 enhancer elements are all located within this broader haplotype and OA association interval. Based on chromatin accessibility and modification in human chondrocytes and developing embryonic limbs, the orthologous human non-coding regions, along with additional regulatory sequences, also appear to be active in relevant tissues and time points during joint development (S8 Fig). Importantly, our functional experiments also reveal that the 3' downstream regions of the mouse *Gdf5* locus, rather than the 5' upstream regions, are capable of driving postnatal expression in knee articular structures (S3 Fig). In addition, it is the Downstream but not Upstream BAC that is capable of fully restoring articular structures and restoring knee ligaments in *Gdf5* mutant mice (Fig 5). Many common variants are located within the syntenic downstream region of the human *GDF5* locus, including common variants found in the human orthologs of the R3, R4, and, R5 enhancers (S4 Table), and likely in sequences that remain unidentified by our approach, including sequences that are located even further upstream or downstream of the gene. While humans may have additional *GDF5* enhancers not yet identified in the mouse studies, the human orthologs of mouse *Gdf5* enhancers are a promising place to look for possible functional non-coding mutations that may alter *GDF5* expression, joint structures, and disease risk in human populations, including changes that are specific to particular subsets of joints in the skeleton.

## Materials and Methods

### Ethics statement

All experiments performed on adult, fetal, and embryonic mice including euthanasia have been approved by the Stanford University Institutional Animal Care and Use Committee (IACUC) and were performed in accordance with Stanford Administrative Panel on Laboratory Animal Care Guidelines (approved protocol 10665), in facilities certified by the American Association of Laboratory Animal Science. No human subjects were utilized in this study. No field permits were required, granted, or utilized for this study.

### BAC modifications

The Upstream *Gdf5*-BAC was isolated from a Research Genetics 129Sv BAC library by PCR screening with specific primers from the *Gdf5* 3'UTR (5'-CGACTCTGCCAACAACGTGG-3' and 5'-CACCTTTCCTGAGCCCCAGG-3'). The size and orientation of the BAC inserts were determined using restriction mapping, pulse-field gel electrophoresis, and Southern analysis. The Downstream BAC RP23-316K12 was identified by searching published mouse BAC-end sequences for those that extend further downstream of the first Upstream BAC.

The two BACs were modified as described previously [93]. A *Gdf5* targeting cassette was made by inserting 5' and 3' homology arms into a recombination vector pIRES-βGeo-Ftet, which contains an IRES-βGeo cassette and a tetracycline resistance cassette flanked by FRT sites. The PCR primers for amplifying the homology arms were (5' Arm: forward with NheI linker 5'-GGATTGCTAGCTATTCATCGACTCTGCCAACAACGTGG-3' and reverse with XhoI linker 5'-GGATTCTCGAGTAAGCAGCTTCACAGGCTCTCTGTTAC-3'. 3' Arm: forward with SpeI linker 5'-GCATGACTAGTGCTGCTGCCCGAAGTTTCCTGG-3' and reverse with NotI linker 5'-GGATTGCGGCCGCTAAAGAACACCTTTCCTGAGCCCCAGG-3'). BAC modifications were carried out by homologous recombination with the targeting cassette in EL250 bacteria strain containing the original *Gdf5*-BACs. Successfully targeted BACs were verified by sequencing, and contained an IRES-βGeo cassette inserted within the 3'UTR of the gene, 747 bp downstream of the *Gdf5* stop codon. The tetracycline resistance gene was subsequently removed by induction of Flpe recombinase.

### Hsp68lacZ plasmid constructions

Evolutionarily conserved non-coding regions (ECR) to be tested for enhancer activity were PCR amplified with primers containing NotI restriction sites, and cloned into the p5'NotI-hsp68 *lacZ* expression vector containing a minimal heat shock promoter and the *lacZ* cassette [94]. For constructs where concatenated copies were required, primers containing SfiI sites were used and the PCR products were ligated briefly to form tandem copies before cloning into a modified p5'NotI-hsp68 *lacZ* vector with a SfiI restriction site inserted between two NotI sites upstream of the hsp68 *lacZ* cassette [75]. All primers used to amplify ECRs described in this study are listed in S5 Table.

### Transgenic mice

Transgenic mice were generated by pronuclear injections carried out either by Michael Schoor, the Stanford Transgenic Facility, or Taconic/Xenogen Biosciences. All constructs were linearized and then were purified for microinjection into FVB or C57BL6/CBA F1 fertilized oocytes as previously described [94, 95]. To facilitate analysis of many different constructs, most transgenic embryos were collected at E14.5 for X-gal staining, without further breeding. For each construct, multiple transgenic embryos derived from independent integration events were analyzed, and only patterns seen consistently in 3 or more independent embryos are reported. Please refer to S1 Table for a summary of the number of transient transgenic embryos generated for each construct, and the expression patterns seen. For the Upstream and Downstream BAC constructs, stable transgenic lines were also generated by allowing injected embryos to come to term, and outcrossing to transmit the transgene. Multiple stable lines were used to confirm E14.5 day expression patterns (S1 Table), and to analyze expression and phenotypic rescue in adult mice.

### X-gal staining and sections

Whole mount staining for β-galactosidase activity was performed as described with minor modifications [94]. Embryos were fixed for 45 minutes in fresh 4% PFA in PBS at 4°C, cut in half and then fixed for additional 15 minutes. Fixed embryos were washed 3 times in wash buffer and stained for 16–24 hours in the dark with 1 mg/ml X-gal in staining buffer at room temperature. After staining, embryos were briefly washed in wash buffer and post-fixed in 4% PFA for 5 hours. Times were adjusted accordingly for E17.5 and post-natal specimens. For sectioning, X-gal stained embryos were placed first in sucrose and then embedded in gelatin/sucrose solution and cryo-sectioned at 25 μm. Sections were counterstained with Nuclear Fast Red (Vector labs, #H-3403).

### *In situ* hybridization

Antisense and sense digoxigenin-labeled probes for *in situ* hybridization were generated for *Gdf5*, *Cep250*, and *Uqcc1*. *Gdf5* probes were generated as described [7]. *Cep250* probes were generated by amplifying a 1593bp fragment corresponding to the 3’UTR from mouse genomic DNA using primers 5’- TTGCCAGAAGAAAGAAGAGCTGAGG-3’ and 5’-TTTATTGTCGAAGGGAAGATGAGGG -3’. The fragment was next cloned into pBluescript SK vector, and sense and antisense probes were produced by digesting with EcoRI and transcribing with T3 and T7 RNA polymerase, respectively. *Uqcc1* probes were generated by amplifying a 1074bp fragment corresponding to the 3’UTR from mouse genomic DNA using primers 5’-TTCACTCAGAAACCCCTGTGCTTGG -3’ and 5’- TGCCCAGATGTAATGAGTTACAAGG-3’. The fragment was then cloned into pBluescript SK vector, and sense and antisense probes were produced by digesting with EcoRI and transcribing with T3 and T7 RNA polymerase, respectively. Next, to compare *Gdf5 lacZ* expression to endogenous gene expression for each of these genes, E15 Downstream BAC embryos were harvested, next snap-frozen and then embedded in Tissue-Tek OCT compound. Embedded embryos were then serially sectioned on a cryostat. Adjacent sections were either stained for *lacZ* expression using the X-gal staining methods described above, or used in standard *in situ* hybridization protocols as described [96].

### Phenotypic rescue experiments

Three independent mouse lines per Upstream BAC or Downstream BAC were each crossed to animals homozygous for *bp*^*J*^ or *bp*^*3J*^ *brachypodism* alleles, respectively. The *bp*^*J*^ mutation occurred spontaneously in A/J mice, and *bp*^*3J*^ in BALB/cJ. Both mutations result in frameshifts and premature translational termination in the *Gdf5* open reading frame [7]. *Gdf5*-BAC/+; *bp*/+ animals were crossed to non-transgenic *bp/bp* mice, and progeny were genotyped for the *lacZ* transgene and *brachypodism* mutations in separate PCR reactions. The primers used for *lacZ* genotyping were: 5'-TTTCCATGTTGCCACTCGC-3' and 5'-AACGGCTTGCCGTTCAGCA-3'. The *brachypodism* mutations were genotyped with primers that amplify the endogenous *Gdf5* locus but not the *Gdf5*-BAC transgene (due to the presence of the IRES-βGeo insertion; forward 5'-ACCTGGAACTCATCTGCACTGTG-3' and reverse 5'-TGGGAAACAGTTTATACCTGAGG-3').

Eight-week old female *Gdf5*-BAC/+; *bp/bp* mice, +/+; *bp/bp* mice, and *+/+*; *bp*/+ mice were stained with Alcian blue (cartilage) and Alizarin red (bone) and cleared as described [97]. Specifically, the skin and visceral organs were removed and the animals were fixed in 95% ethanol. The specimens were then stained in a mixture of Alcian blue, acetic acid, and ethanol. After several washes in 95% ethanol, the samples were cleared in KOH and then stained by Alizarin red in KOH solution. The skeletons were stored and photographed in glycerol. Knees and hindpaws were examined and imaged under a light microscope for joint morphology and the presence/absence of the cruciate ligaments. For radiographic analysis, intact hindlimbs were positioned laterally on Kodak radiographic film and placed in a Faxitron X-ray system at 18kV and 0.3mA, for 22 seconds. For each group of animals, homozygous *bp* animals without the BAC, homozygous *bp* animals with the BAC and heterozygous *bp* animals without the BAC, n = 8.

### Comparative sequence analysis

Sequence data for different species was obtained by searching publicly available nucleotide databases at NCBI (http://www.ncbi.nlm.nih.gov/BLAST/). Evolutionarily conserved non-coding sequences were identified using global sequence alignment programs including:

PipMaker (http://bio.cse.psu.edu/pipmaker),

VISTA (formerly at http://genome.lbl.gov/vista/index.shtml, [98]), and

LAGAN (http://lagan.stanford.edu/lagan_web/index.shtml, [99]).

### Predicted transcription factor binding sites

To find upstream transcription factors predicted to bind to R2 regulatory sequences, we used Vista with MatInspector, and UNIPROBE. rVista uses the TRANSFAC database to look for potential binding sites that are highly conserved across sequences [38, 39]. MatInspector of the Genomatix suite (http://www.genomatix.de) uses a large library of matrix descriptions for transcription factor binding sites to find corresponding matches in a target sequence [37, 40]. The UNIPROBE database (http://the_brain.bwh.harvard.edu/uniprobe/about.php) is built on replicate experimental measurements of binding affinities between large numbers of expressed transcription factors and all possible 8-mer target oligonucleotides [50, 100]. At the recommended enrichment threshold of 0.4, UNIPROBE identified over 3,000 sites (i.e., specific 8-mer sequences bound by a transcription factor) in mouse and human R2 sequences. The relevant factors, 8-mers (and reverse complements), positions in the R2 sequence, and enrichment scores depicting relative binding affinity for each factor are summarized in S2 Table, sheets 1–2.

Lists of potential upstream regulators were intersected with expression and phenotypic data in order to identify those transcription factors also known to be expressed or required in limbs and joints, based on data in VisiGene (http://genome.ucsc.edu/cgi-bin/hgVisigene), Eurexpress (http://www.eurexpress.org/ee/), Genepaint (http://www.genepaint.org/Frameset.html), and the Mouse Genome Informatics expression and phenotypic databases (http://www.informatics.jax.org). *Barx2* displayed overlap with known *Gdf5* expression patterns, specifically at gestational days when R2 enhancer was active [41–44]. *Pitx1* is expressed in hindlimbs and is required for normal development of knees [55]. *Zeb1* is also expressed in joints, and null mice exhibit multiple skeletal defects, including the fusion of the humerus to either radius or ulna [53].

Engineered R2 sequences carrying site-specific mutations in predicted BARX2 binding sites were synthesized by GenScript. S3 Table shows the calculated changes in UNIPROBE enrichment scores for BARX2 binding to wild type and mutated sites. 2X copies of wild type and mutant R2 sequences were cloned into the Hsp68 *lacZ* reporter with appropriate primers and restriction enzyme sites (S5 Table), and used to generate multiple independent E14.5 transgenic mouse embryos for *lacZ* expression analysis as above (S1 Table).

A three-base pair mutation was engineered in the single ZEB1 binding site and the single PITX binding site in the R2d+e region, based on presence and loss of binding sites in MatInspector. Altered R2d+e constructs were synthesized by PCR-sequence overlap extension (PCR-SOEing) [101] using the primers listed in S5 Table. Wild type and mutant constructs were and used to generate E14.5 transgenic mouse embryos as above (S1 Table). Neither binding site mutation produced a noticeable alteration in the limb joint expression patterns driven by the unaltered R2d+e construct.

## Supporting Information

S1 Table Summary of expression patterns driven by BACs or smaller constructs in independent E14.5 transgenic embryos. (XLSX)

S2 Table UNIPROBE analysis of transcription factor binding sites in human and mouse R2 sequences. (XLS)

S3 Table UNIPROBE analysis of engineered mutations in mouse R2 sequence. (XLS)

S4 Table Summary of common human polymorphisms that map in non-coding orthologs of mouse *Gdf5* enhancers. (XLSX)

S5 Table Primers used to make hsp68-*lacZ* transgene constructs. (PPT)

S1 Fig Upstream BAC transgenic embryos recapitulate many sites of endogenous *Gdf5* expression in axial joints and connective tissues. In all panels the ventral side is left and anterior is at top. **a**, Side view of E14.5 Upstream BAC transgenic embryo showing *lacZ* expression in numerous anatomical locations (a, ankle; e, elbow joint; h, hip joint; k, knee joint; me, middle ear; r, ribs; s, shoulder joint; w, wrist) **b**, Medial view of bisected embryo as in a, showing *lacZ* expression internally (cp, choroid plexus; l, larynx; n, neck joint; st, sternum; t, tail ligament; vc, vertebral column). **c**, *lacZ* expression in tooth buds. **d**, *lacZ* expression in sternal joints. **e**, Endogenous *Gdf5* expression in tooth bud (white arrowhead) and sternal joints (black arrowheads). **f**, *lacZ* expression in joint between basioccipital bone (b) and atlas (at). In f, the joint between the bodies of the atlas and axis is out of the plane of section (ax* = transverse process of the axis). **g**, Endogenous *Gdf5* expression in basioccipital-atlas joint and atlas-axis joint (ax = axis). **h**, *lacZ* expression in rib-vertebral joints (black arrowheads) and intervertebral joints (white arrowheads). **i**, Endogenous *Gdf5* expression similar to *lacZ* expression shown in h. c, d, f, and h are sagittal cryosections of E14.5 Upstream BAC transgenic embryos stained by X-gal and counterstained with neutral red. e, g, and i show *in situ* hybridization with antisense *Gdf5* probe to sagittal cryosections of nontransgenic E14.5 embryos.
(TIF)

S2 Fig Expression of Upstream BAC transgene in adult mice. **a**, Cleared foot skeleton from a transgenic Upstream BAC positive adult 2 month old mouse, stained with Alizarin red and with X-gal, showing persistence of *lacZ* expression in joints. **b**, Magnified view of a metacarpal-phalangeal joint showing *lacZ* expression on the articular surface. **c**, Section through joint in b showing *lacZ* expression in the superficial chondrocytes of the articular cartilage. **d**, Magnified view of vertebral articulation of Upstream BAC transgenic in a 2 month old adult mouse, stained as in a. Note *lacZ* expression at the sites of articulation. **e**, *lacZ* expression at the insertion site (arrowhead) of the Achilles tendon on the calcaneus.
(TIF)

S3 Fig Comparison of Upstream and Downstream BAC expression patterns in pre- and post-natal knee structures. Xgal stained knee joints of mice harboring either the Upstream BAC (**A**-**D**)) or Downstream BAC (**E**-**H**) at late gestational stages (E17.5 embryos) (**A**, **B**, **E**, **F**) or post-natal 3 month old adults (**C**, **D**, **G**, **H**). At E17.5, regulatory elements within the Upstream BAC (**A**, **B**) drove moderate expression in the cruciate ligaments (Cr) as well as the articular surfaces of the femur and tibia (denoted with an asterisk, *), whereas sequences within the Downstream BAC (**E**, **F**) drove strong expression in these domains, along with additional expanded locations such as the articular capsule (AC) and the medial and lateral meniscus (Me). At post-natal 3 months, end-on views are shown of the exposed articular surfaces of the femur (**C**, **G**) and of the tibia (**D**, **H**). Note that regulatory sequences within the Downstream BAC continue to drive strong expression in the articular surfaces of the femur (i.e., patella groove, PG; femoral condyle, FC) and the tibia (i.e., tibial platform, TP), with expression persisting in the meniscus (Me, lateral meniscus removed to expose TP) and collateral ligaments (CL) (panels **G**, **H**). These latter post-natal patterns were not observed in mice carrying the Upstream BAC reporter (panels **C**, **D**).
(TIF)

S4 Fig Expression patterns driven by R2, R3+R4+R5, and R4 constructs. (**A**) Four evolutionarily conserved elements (red boxes) within the Upstream (yellow) and Downstream (green) BACs were identified via a comparison of mouse, chicken, and human sequence conservation. Pink peaks represent conserved non-coding sequence showing at least 70% nucleotide identity over a 300 bp window, while blue peaks reflect conservation in coding sequence. (**B**) First row: representative transgenic embryos showing *lacZ* expression patterns driven by R2, R3+R4+R5, and R4 constructs. Second row: forepaw expression in these embryos. Third and fourth rows: serial sections of elbow joints. Note that R2, R3+R4+R5, and R4 drove *lacZ* expression in proximal synovial joints, such as shoulder (s), elbow (e), and knee (k), as well as distal joints of wrist (w), ankle (a), and digit joints (d), although for the R2 element, digital expression was inconsistent (i.e., less than half of *lacZ* positive embryos exhibited this expression pattern). R3+R4+R5 drove strong digit (d or white arrowhead) and interdigital space (white arrow) *lacZ* expression, while R4 only drove joint expression (white arrowhead). In contrast to R2, which only drove *lacZ* expression in humeroradial (h-r) joint, R3+R4+R5 drove expression in both humeroradial (h-r) and the humeroulnar (h-u) joints. R4 expression in the elbow was similar to that of R3+R4+R5.
(TIF)

S5 Fig Mutations in predicted PITX1 and ZEB1 binding sites do not alter R2d+e expression patterns. (**A**) Schematic representation of R2 sub-regions R2d and R2e, and the locations (asterisk, *) of two predicted transcription factor binding sites for PITX1 and ZEB1. A nine-way species alignment reveals the high degree of sequence conservation in and around each predicted binding site (underlined). Red bases show the bases mutated to produce the ΔPitx1 and ΔZeb1 enhancer constructs. (**B**) Comparison of transgenic embryos carrying the wild type (R2d+e), or mutant enhancer constructs (ΔPitx1 and ΔZeb1). All constructs drove similar *lacZ* expression in the proximal limb joints of shoulder (s), elbow (e), hip, and knee (k). Expression patterns outside the limb were not consistent for either the R2d+e construct or the constructs with mutant binding sites.
(TIF)

S6 Fig Comparison of expression patterns of *Gdf5* and neighboring genes *Cep250* and *Uqcc1*. Panels show near adjacent sections of developing E15 hindlimbs (top row, **A**-**D**) and knees (bottom row, **E**-**H**), hybridized with probes for the indicated genes, or stained for *Gdf5* Downstream BAC*-LacZ* expression. (**A**, **E**) Expression of *Gdf5* in joints of the hindlimb and knee. (**B**, **F**) *lacZ* activity driven by the Downstream BAC shows expression in the same joint structures as the endogenous *Gdf5* gene. In contrast, (**C**, **G**) the *Cep250* gene shows weak expression in muscle tissue of the limb (red arrowheads), and (**D**, **H**) the *Uqcc1* shows little or no concentrated staining in particular structures of the hindlimb at this stage. Abbreviations: ankle (a), femur (f), fibula (fi), and tibia (t).
(TIF)

S7 Fig Osteoarthritis-associated risk alleles at 5'UTR positions do not alter qualitative patterns driven by human *GDF5* constructs. (**A**) Adapted hg19 UCSC browser screenshot showing the 5' region of the human *GDF5* locus, including the locations of two common human SNPs (rs143383 and rs143384) found in the 5'UTR. Derived alleles at these positions ("T, T") have previously been associated with increased risk of osteoarthritis [27]. (**B**) We generated two Hsp68 *lacZ* expression constructs containing an identical 1468 base pair region (hg19, chr20: 34,025,720–34,027,187 corresponding to +367 to -1,101 of the *GDF5* promoter region [27]) that differed only by having ancestral ("C, C") or derived risk ("T, T”) alleles at the rs143383 and rs143384 positions. Both constructs drove similar joint expression patterns in E14.5 day transgenic embryos. Abbreviations: shoulder (s), elbow (e), and knee (k).
(TIF)

S8 Fig Evolutionary conservation and chromatin mark labeling of regulatory sequences. UCSC Genome Browser view of a 130 kb region surrounding the human *GDF*5 locus on chromosome 20 (genome version hg19). This view highlights the genomic locations of the coding exons of *GDF5* and *UQCC* (track: UCSC Genes); the position of five human sequences orthologous to the functionally defined mouse enhancers R1-R5 (track: *GDF5* Regulatory Elements); and larger patterns of evolutionary sequence conservation based on 100 sequenced vertebrates (Tracks: 100 vertebrates Basewise Conservation by PhyloP; Multiz Alignments of 100 Vertebrates). Histone marks from chromatin immunoprecipitation (ChIP-seq) tracks in human and mouse tissues are shown as separate tracks. Data from Cotney et al (2013) [102] shows the locations H3K27ac peaks (active enhancers) in developing human and mouse limbs at four embryonic timepoints (E33, E41, E44, and E47) corresponding to equivalent mouse (mm9) gestational days (E10.5, E11.5, E12.5, and E13.5). Note that the downstream enhancer regions R3-R5 show peaks of H3k27ac signal at times of active joint formation in both humans and mice (Hg19E44+Hg19E47; Mm9E12.5+Mm9E13.5), but not at early stages when joints have not begun to form (Hg19E33+Hg19E41; Mm9E10.5+Mm9E11.5). A second experimental data track, acquired from the Roadmap Epigenomics Project (see text) [103], consists of significant H3K27ac peaks in chondrocytes derived from adult human bone marrow. Note that H3K27ac peaks are seen over R1-R5 in human chondrocytes from four separate donors.
(TIF)
