# Supplementary material for: Heads, Shoulders, Elbows, Knees, and Toes: Modular Gdf5 Enhancers Control Different Joints in the Vertebrate Skeleton
Source: PLoS Genet. 2016 Nov 30;12(11):e1006454. doi: 10.1371/journal.pgen.1006454 (PMC5130176; doi:10.1371/journal.pgen.1006454)
Supplement: S5 Table — (PPT) [file pgen.1006454.s005.ppt]

## Slide 1
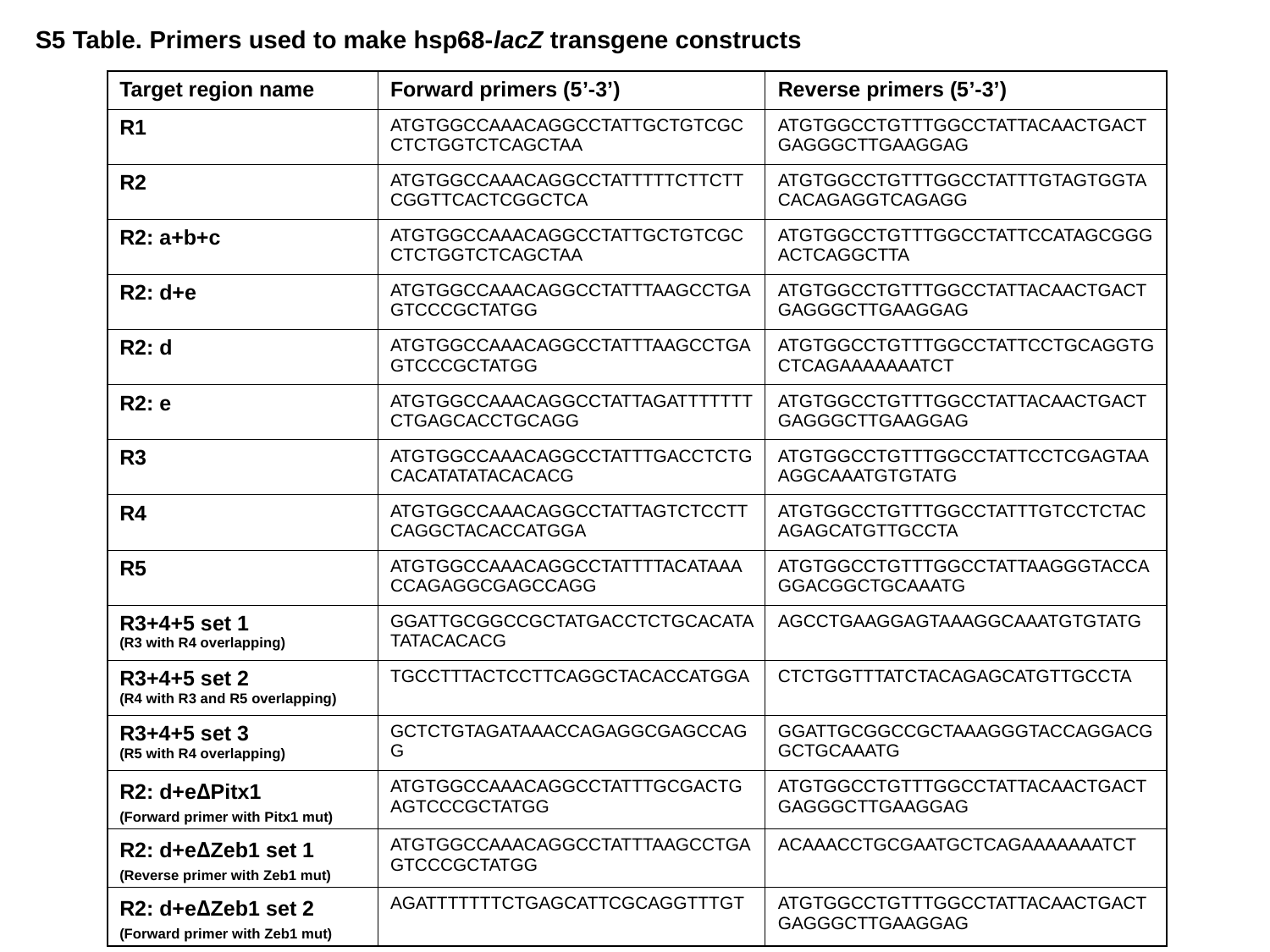

S5 Table. Primers used to make hsp68-lacZ transgene constructs
| Target region name | Forward primers (5’-3’) | Reverse primers (5’-3’) |
| --- | --- | --- |
| R1 | ATGTGGCCAAACAGGCCTATTGCTGTCGCCTCTGGTCTCAGCTAA | ATGTGGCCTGTTTGGCCTATTACAACTGACTGAGGGCTTGAAGGAG |
| R2 | ATGTGGCCAAACAGGCCTATTTTTCTTCTTCGGTTCACTCGGCTCA | ATGTGGCCTGTTTGGCCTATTTGTAGTGGTACACAGAGGTCAGAGG |
| R2: a+b+c | ATGTGGCCAAACAGGCCTATTGCTGTCGCCTCTGGTCTCAGCTAA | ATGTGGCCTGTTTGGCCTATTCCATAGCGGGACTCAGGCTTA |
| R2: d+e | ATGTGGCCAAACAGGCCTATTTAAGCCTGAGTCCCGCTATGG | ATGTGGCCTGTTTGGCCTATTACAACTGACTGAGGGCTTGAAGGAG |
| R2: d | ATGTGGCCAAACAGGCCTATTTAAGCCTGAGTCCCGCTATGG | ATGTGGCCTGTTTGGCCTATTCCTGCAGGTGCTCAGAAAAAAATCT |
| R2: e | ATGTGGCCAAACAGGCCTATTAGATTTTTTTCTGAGCACCTGCAGG | ATGTGGCCTGTTTGGCCTATTACAACTGACTGAGGGCTTGAAGGAG |
| R3 | ATGTGGCCAAACAGGCCTATTTGACCTCTGCACATATATACACACG | ATGTGGCCTGTTTGGCCTATTCCTCGAGTAAAGGCAAATGTGTATG |
| R4 | ATGTGGCCAAACAGGCCTATTAGTCTCCTTCAGGCTACACCATGGA | ATGTGGCCTGTTTGGCCTATTTGTCCTCTACAGAGCATGTTGCCTA |
| R5 | ATGTGGCCAAACAGGCCTATTTTACATAAACCAGAGGCGAGCCAGG | ATGTGGCCTGTTTGGCCTATTAAGGGTACCAGGACGGCTGCAAATG |
| R3+4+5 set 1 (R3 with R4 overlapping) | GGATTGCGGCCGCTATGACCTCTGCACATATATACACACG | AGCCTGAAGGAGTAAAGGCAAATGTGTATG |
| R3+4+5 set 2 (R4 with R3 and R5 overlapping) | TGCCTTTACTCCTTCAGGCTACACCATGGA | CTCTGGTTTATCTACAGAGCATGTTGCCTA |
| R3+4+5 set 3 (R5 with R4 overlapping) | GCTCTGTAGATAAACCAGAGGCGAGCCAGG | GGATTGCGGCCGCTAAAGGGTACCAGGACGGCTGCAAATG |
| R2: d+eΔPitx1 (Forward primer with Pitx1 mut) | ATGTGGCCAAACAGGCCTATTTGCGACTGAGTCCCGCTATGG | ATGTGGCCTGTTTGGCCTATTACAACTGACTGAGGGCTTGAAGGAG |
| R2: d+eΔZeb1 set 1 (Reverse primer with Zeb1 mut) | ATGTGGCCAAACAGGCCTATTTAAGCCTGAGTCCCGCTATGG | ACAAACCTGCGAATGCTCAGAAAAAAATCT |
| R2: d+eΔZeb1 set 2 (Forward primer with Zeb1 mut) | AGATTTTTTTCTGAGCATTCGCAGGTTTGT | ATGTGGCCTGTTTGGCCTATTACAACTGACTGAGGGCTTGAAGGAG |
