# Supplementary figures and images for: Heads, Shoulders, Elbows, Knees, and Toes: Modular Gdf5 Enhancers Control Different Joints in the Vertebrate Skeleton
Source: PLoS Genet. 2016 Nov 30;12(11):e1006454. doi: 10.1371/journal.pgen.1006454 (PMC5130176; doi:10.1371/journal.pgen.1006454)

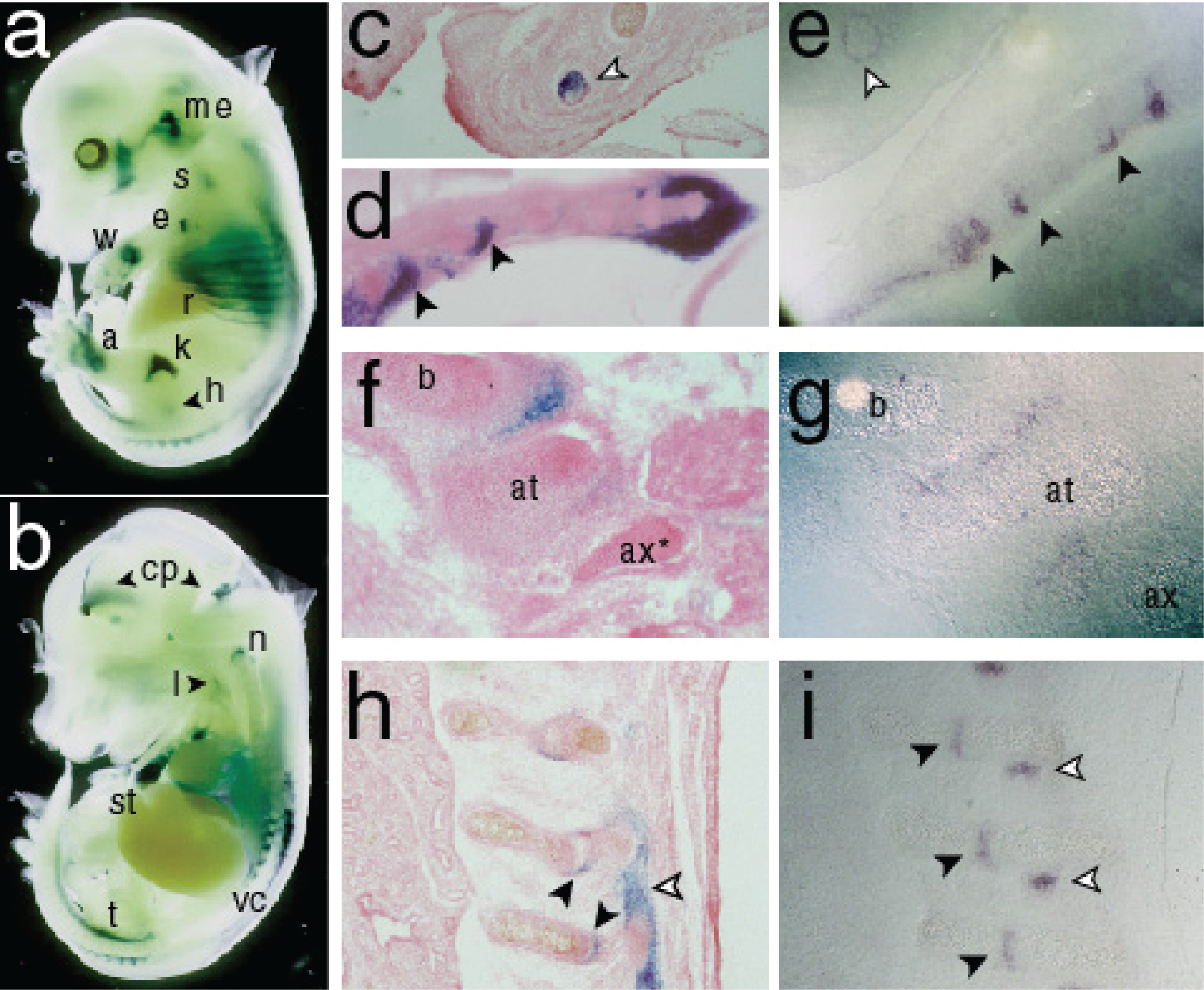

Supplement: S1 Fig — In all panels the ventral side is left and anterior is at top. a, Side view of E14.5 Upstream BAC transgenic embryo showing lacZ expression in numerous anatomical locations (a, ankle; e, elbow joint; h, hip joint; k, knee joint; me, middle ear; r, ribs; s, shoulder joint; w, wrist) b, Medial view of bisected embryo as in a, showing lacZ expression internally (cp, choroid plexus; l, larynx; n, neck joint; st, sternum; t, tail ligament; vc, vertebral column). c, lacZ expression in tooth buds. d, lacZ expression in sternal joints. e, Endogenous Gdf5 expression in tooth bud (white arrowhead) and sternal joints (black arrowheads). f, lacZ expression in joint between basioccipital bone (b) and atlas (at). In f, the joint between the bodies of the atlas and axis is out of the plane of section (ax* = transverse process of the axis). g, Endogenous Gdf5 expression in basioccipital-atlas joint and atlas-axis joint (ax = axis). h, lacZ expression in rib-vertebral joints (black arrowheads) and intervertebral joints (white arrowheads). i, Endogenous Gdf5 expression similar to lacZ expression shown in h. c, d, f, and h are sagittal cryosections of E14.5 Upstream BAC transgenic embryos stained by X-gal and counterstained with neutral red. e, g, and i show in situ hybridization with antisense Gdf5 probe to sagittal cryosections of nontransgenic E14.5 embryos. (TIF) [file pgen.1006454.s006.tif]

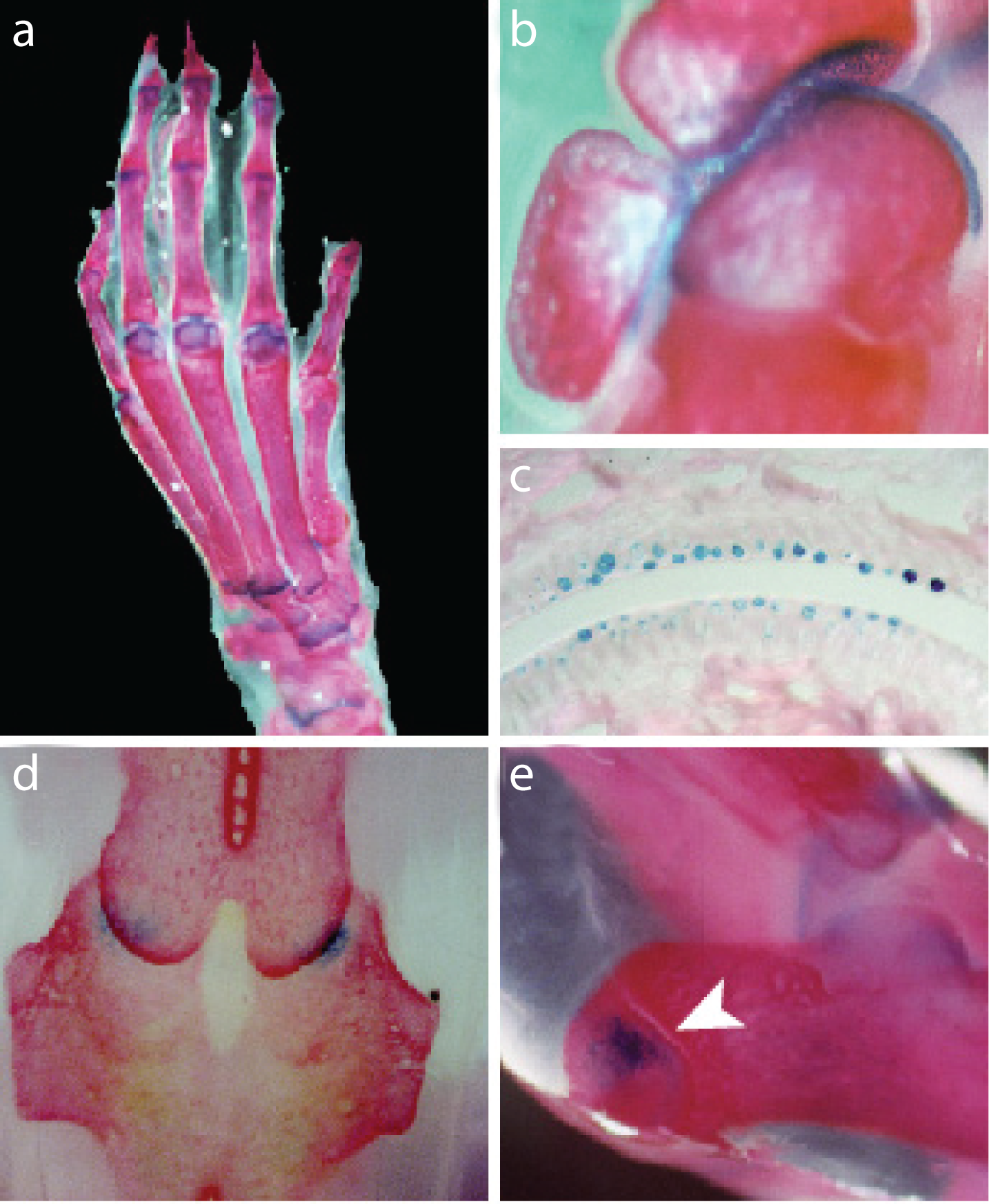

Supplement: S2 Fig — a, Cleared foot skeleton from a transgenic Upstream BAC positive adult 2 month old mouse, stained with Alizarin red and with X-gal, showing persistence of lacZ expression in joints. b, Magnified view of a metacarpal-phalangeal joint showing lacZ expression on the articular surface. c, Section through joint in b showing lacZ expression in the superficial chondrocytes of the articular cartilage. d, Magnified view of vertebral articulation of Upstream BAC transgenic in a 2 month old adult mouse, stained as in a. Note lacZ expression at the sites of articulation. e, lacZ expression at the insertion site (arrowhead) of the Achilles tendon on the calcaneus. (TIF) [file pgen.1006454.s007.tif]

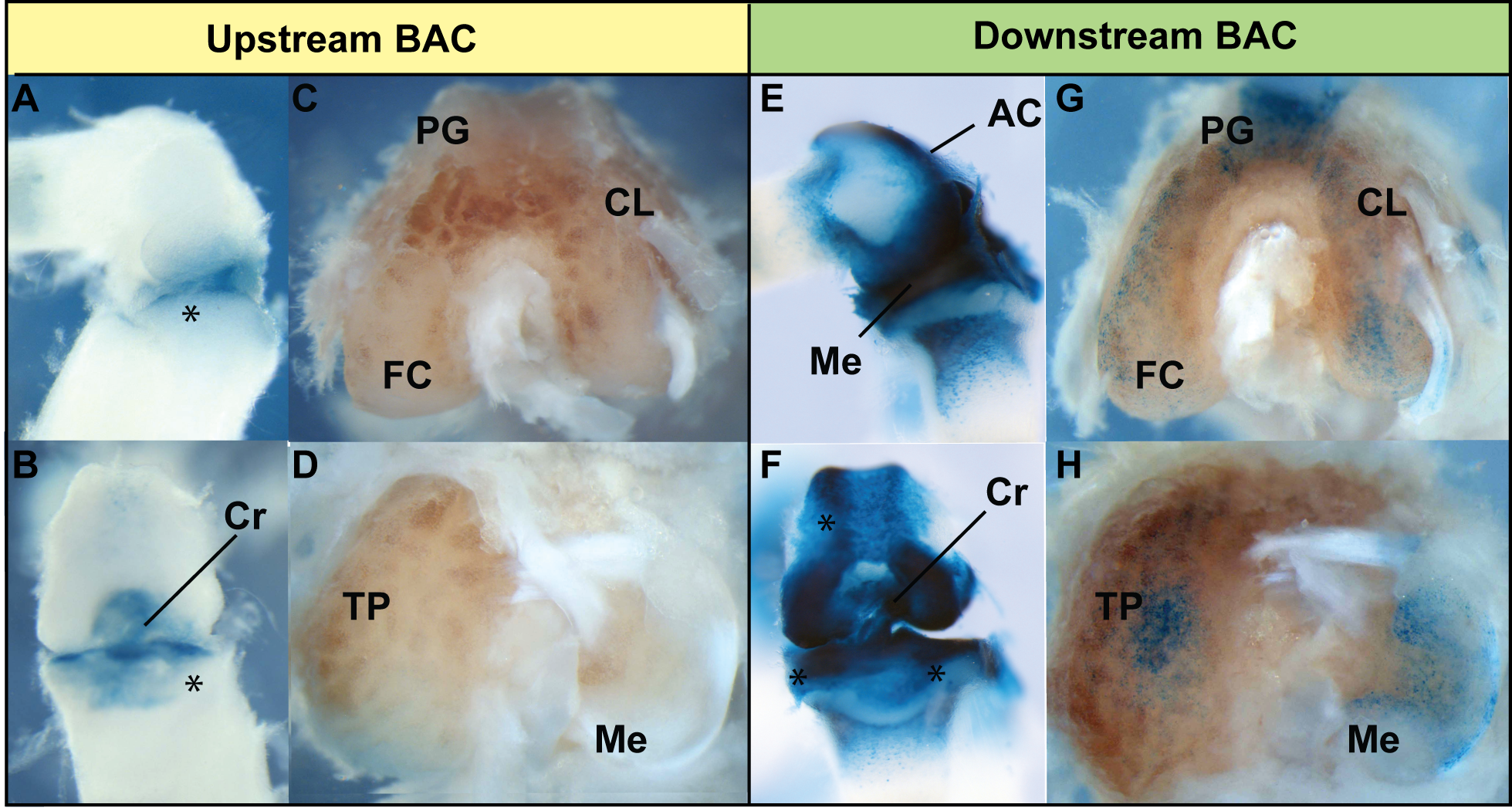

Supplement: S3 Fig — Xgal stained knee joints of mice harboring either the Upstream BAC (A-D)) or Downstream BAC (E-H) at late gestational stages (E17.5 embryos) (A, B, E, F) or post-natal 3 month old adults (C, D, G, H). At E17.5, regulatory elements within the Upstream BAC (A, B) drove moderate expression in the cruciate ligaments (Cr) as well as the articular surfaces of the femur and tibia (denoted with an asterisk, *), whereas sequences within the Downstream BAC (E, F) drove strong expression in these domains, along with additional expanded locations such as the articular capsule (AC) and the medial and lateral meniscus (Me). At post-natal 3 months, end-on views are shown of the exposed articular surfaces of the femur (C, G) and of the tibia (D, H). Note that regulatory sequences within the Downstream BAC continue to drive strong expression in the articular surfaces of the femur (i.e., patella groove, PG; femoral condyle, FC) and the tibia (i.e., tibial platform, TP), with expression persisting in the meniscus (Me, lateral meniscus removed to expose TP) and collateral ligaments (CL) (panels G, H). These latter post-natal patterns were not observed in mice carrying the Upstream BAC reporter (panels C, D). (TIF) [file pgen.1006454.s008.tif]

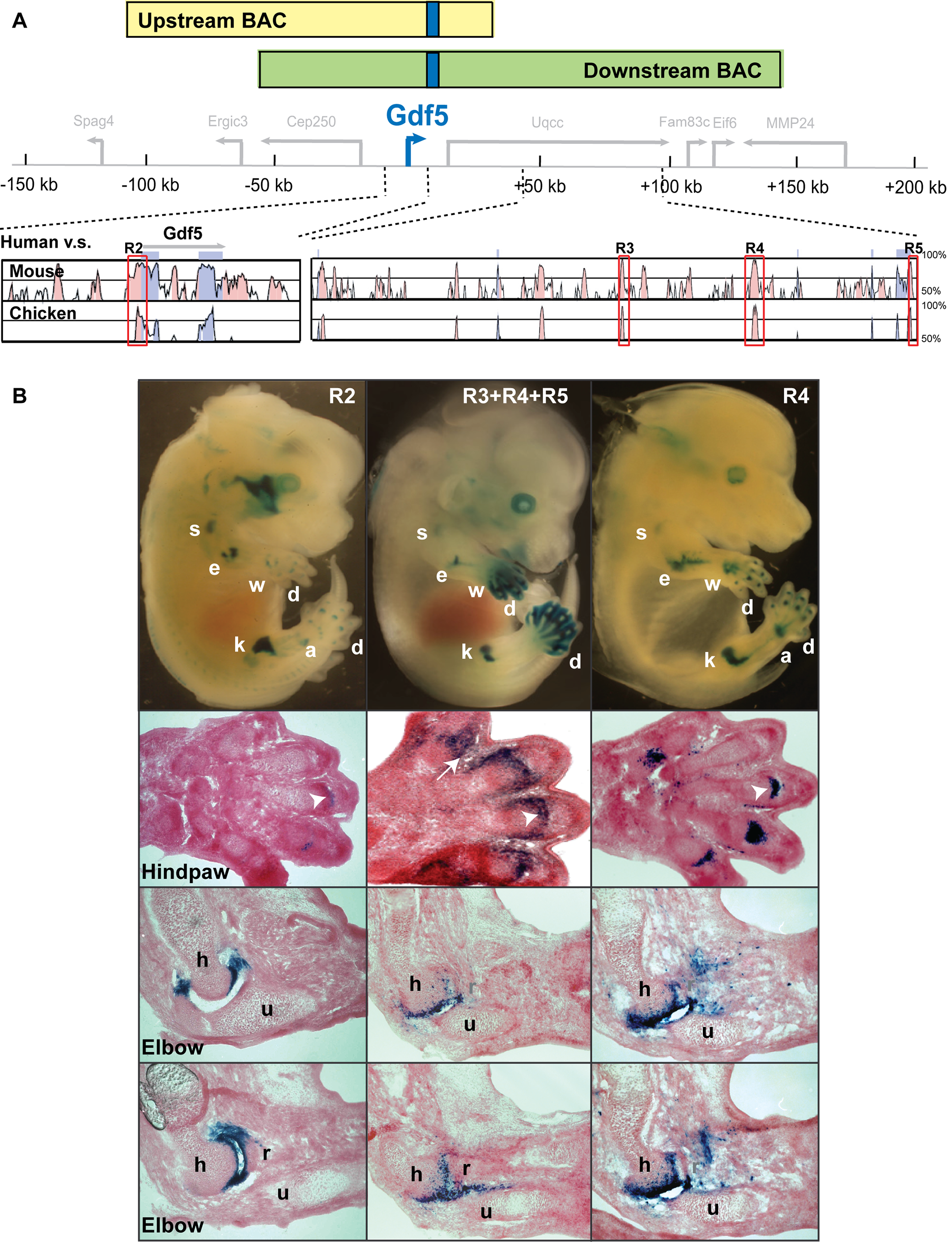

Supplement: S4 Fig — (A) Four evolutionarily conserved elements (red boxes) within the Upstream (yellow) and Downstream (green) BACs were identified via a comparison of mouse, chicken, and human sequence conservation. Pink peaks represent conserved non-coding sequence showing at least 70% nucleotide identity over a 300 bp window, while blue peaks reflect conservation in coding sequence. (B) First row: representative transgenic embryos showing lacZ expression patterns driven by R2, R3+R4+R5, and R4 constructs. Second row: forepaw expression in these embryos. Third and fourth rows: serial sections of elbow joints. Note that R2, R3+R4+R5, and R4 drove lacZ expression in proximal synovial joints, such as shoulder (s), elbow (e), and knee (k), as well as distal joints of wrist (w), ankle (a), and digit joints (d), although for the R2 element, digital expression was inconsistent (i.e., less than half of lacZ positive embryos exhibited this expression pattern). R3+R4+R5 drove strong digit (d or white arrowhead) and interdigital space (white arrow) lacZ expression, while R4 only drove joint expression (white arrowhead). In contrast to R2, which only drove lacZ expression in humeroradial (h-r) joint, R3+R4+R5 drove expression in both humeroradial (h-r) and the humeroulnar (h-u) joints. R4 expression in the elbow was similar to that of R3+R4+R5. (TIF) [file pgen.1006454.s009.tif]

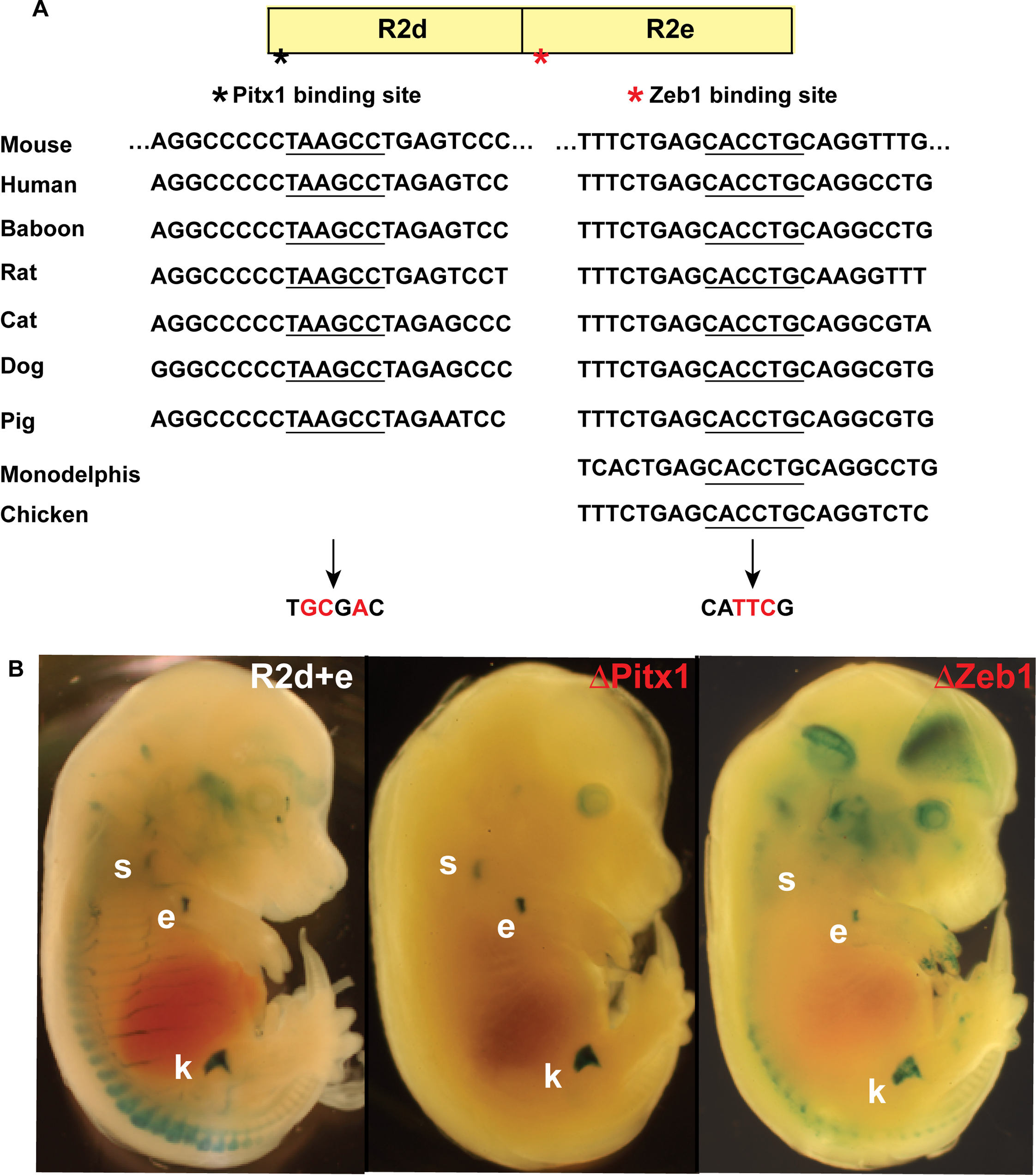

Supplement: S5 Fig — (A) Schematic representation of R2 sub-regions R2d and R2e, and the locations (asterisk, *) of two predicted transcription factor binding sites for PITX1 and ZEB1. A nine-way species alignment reveals the high degree of sequence conservation in and around each predicted binding site (underlined). Red bases show the bases mutated to produce the ΔPitx1 and ΔZeb1 enhancer constructs. (B) Comparison of transgenic embryos carrying the wild type (R2d+e), or mutant enhancer constructs (ΔPitx1 and ΔZeb1). All constructs drove similar lacZ expression in the proximal limb joints of shoulder (s), elbow (e), hip, and knee (k). Expression patterns outside the limb were not consistent for either the R2d+e construct or the constructs with mutant binding sites. (TIF) [file pgen.1006454.s010.tif]

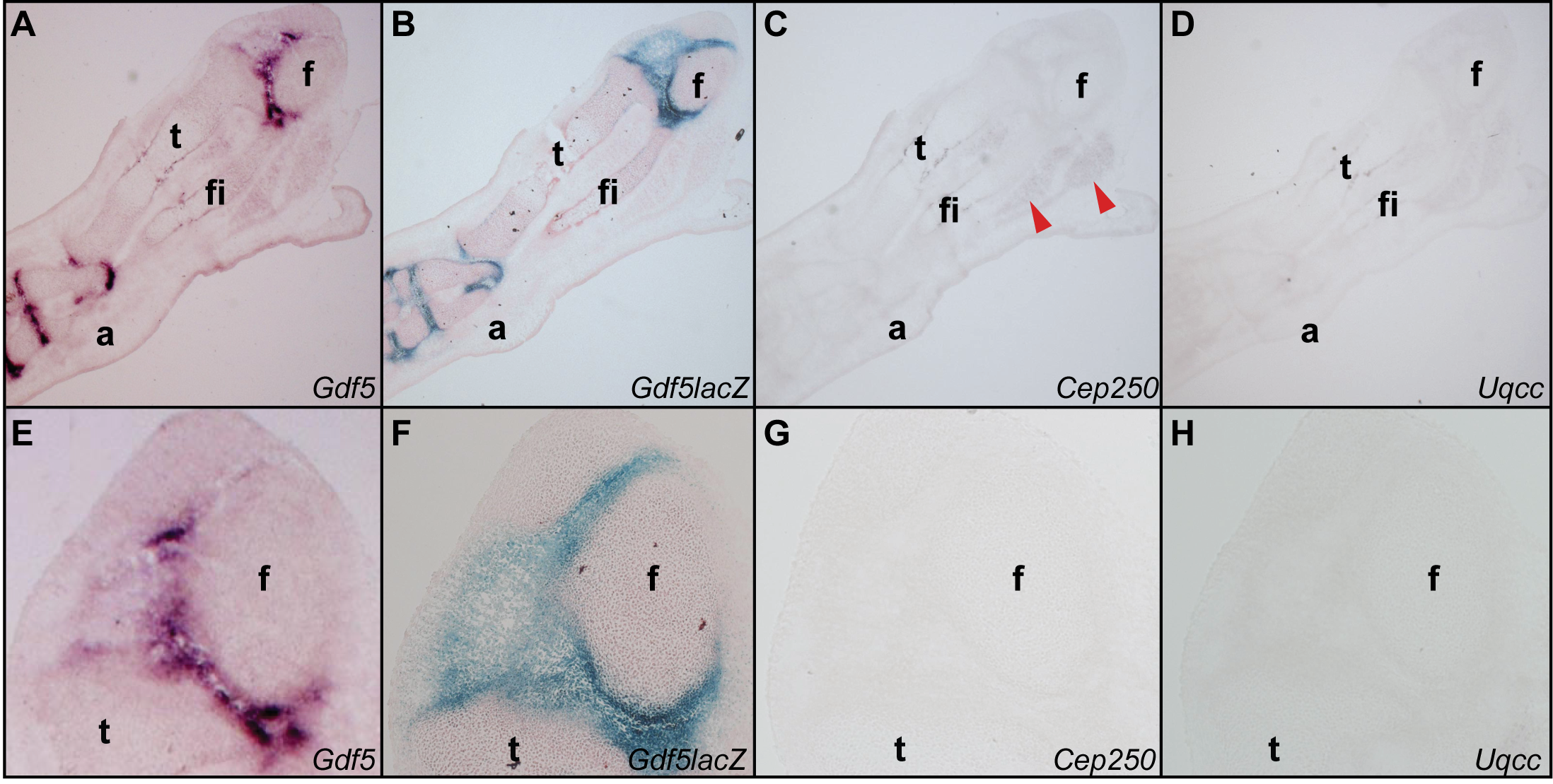

Supplement: S6 Fig — Panels show near adjacent sections of developing E15 hindlimbs (top row, A-D) and knees (bottom row, E-H), hybridized with probes for the indicated genes, or stained for Gdf5 Downstream BAC-LacZ expression. (A, E) Expression of Gdf5 in joints of the hindlimb and knee. (B, F) lacZ activity driven by the Downstream BAC shows expression in the same joint structures as the endogenous Gdf5 gene. In contrast, (C, G) the Cep250 gene shows weak expression in muscle tissue of the limb (red arrowheads), and (D, H) the Uqcc1 shows little or no concentrated staining in particular structures of the hindlimb at this stage. Abbreviations: ankle (a), femur (f), fibula (fi), and tibia (t). (TIF) [file pgen.1006454.s011.tif]

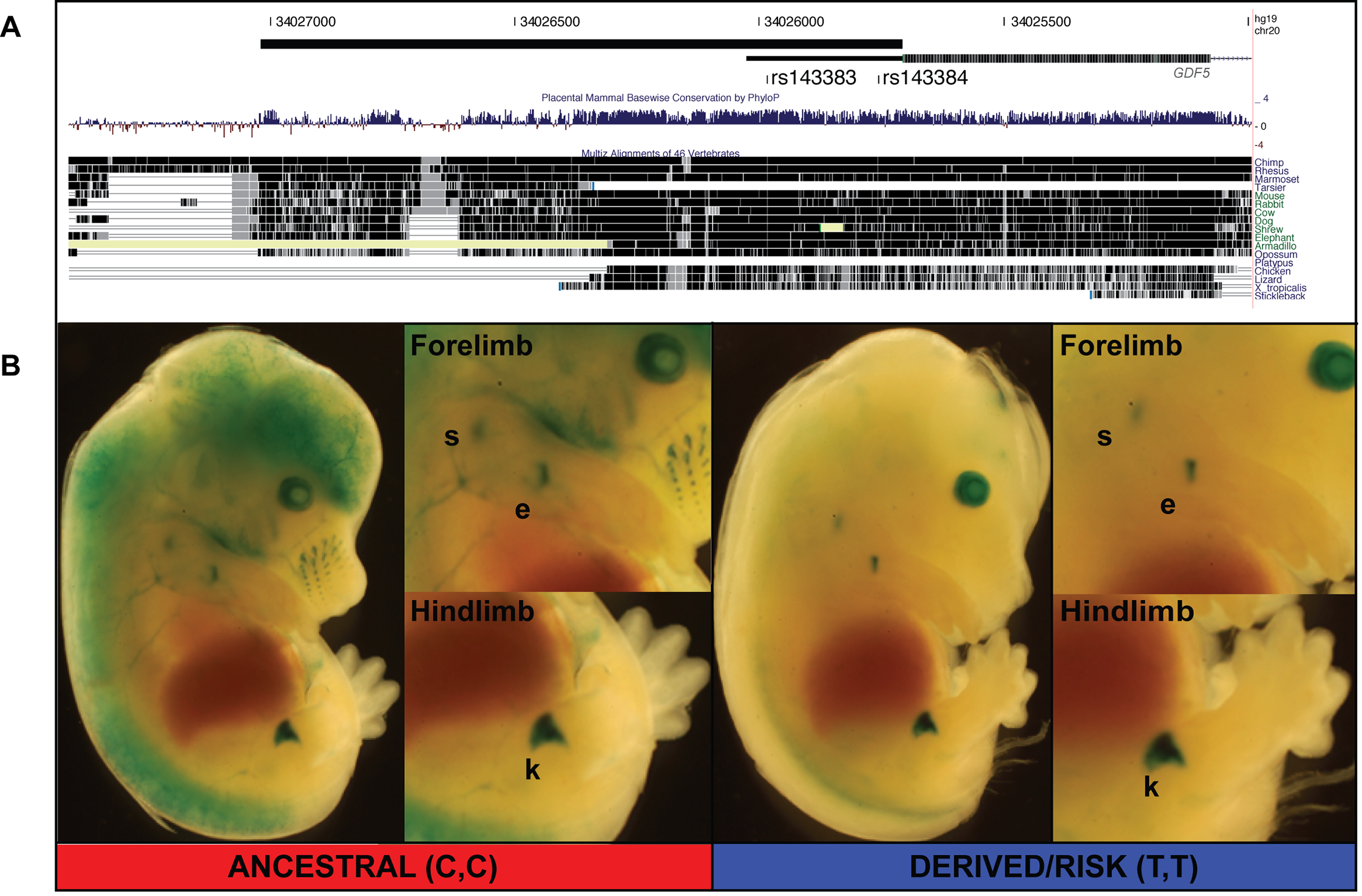

Supplement: S7 Fig — (A) Adapted hg19 UCSC browser screenshot showing the 5' region of the human GDF5 locus, including the locations of two common human SNPs (rs143383 and rs143384) found in the 5'UTR. Derived alleles at these positions ("T, T") have previously been associated with increased risk of osteoarthritis [27]. (B) We generated two Hsp68 lacZ expression constructs containing an identical 1468 base pair region (hg19, chr20: 34,025,720–34,027,187 corresponding to +367 to -1,101 of the GDF5 promoter region [27]) that differed only by having ancestral ("C, C") or derived risk ("T, T”) alleles at the rs143383 and rs143384 positions. Both constructs drove similar joint expression patterns in E14.5 day transgenic embryos. Abbreviations: shoulder (s), elbow (e), and knee (k). (TIF) [file pgen.1006454.s012.tif]

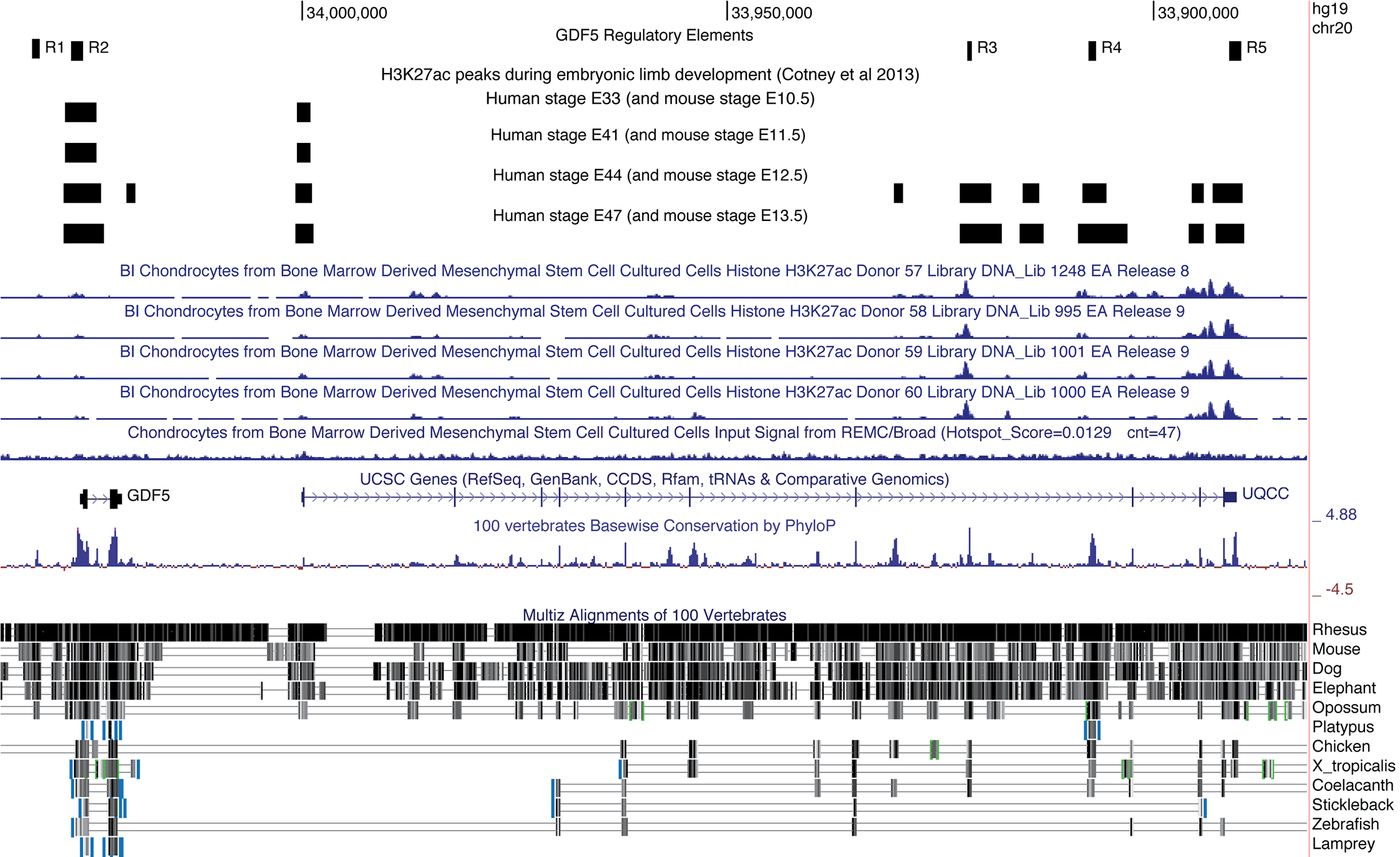

Supplement: S8 Fig — UCSC Genome Browser view of a 130 kb region surrounding the human GDF5 locus on chromosome 20 (genome version hg19). This view highlights the genomic locations of the coding exons of GDF5 and UQCC (track: UCSC Genes); the position of five human sequences orthologous to the functionally defined mouse enhancers R1-R5 (track: GDF5 Regulatory Elements); and larger patterns of evolutionary sequence conservation based on 100 sequenced vertebrates (Tracks: 100 vertebrates Basewise Conservation by PhyloP; Multiz Alignments of 100 Vertebrates). Histone marks from chromatin immunoprecipitation (ChIP-seq) tracks in human and mouse tissues are shown as separate tracks. Data from Cotney et al (2013) [102] shows the locations H3K27ac peaks (active enhancers) in developing human and mouse limbs at four embryonic timepoints (E33, E41, E44, and E47) corresponding to equivalent mouse (mm9) gestational days (E10.5, E11.5, E12.5, and E13.5). Note that the downstream enhancer regions R3-R5 show peaks of H3k27ac signal at times of active joint formation in both humans and mice (Hg19E44+Hg19E47; Mm9E12.5+Mm9E13.5), but not at early stages when joints have not begun to form (Hg19E33+Hg19E41; Mm9E10.5+Mm9E11.5). A second experimental data track, acquired from the Roadmap Epigenomics Project (see text) [103], consists of significant H3K27ac peaks in chondrocytes derived from adult human bone marrow. Note that H3K27ac peaks are seen over R1-R5 in human chondrocytes from four separate donors. (TIF) [file pgen.1006454.s013.tif]
